# Beyond the Warburg Effect: Modeling the Dynamic and Context-Dependent Nature of Tumor Metabolism

**DOI:** 10.3390/cancers17213563

**Published:** 2025-11-03

**Authors:** Pierre Jacquet, Angélique Stéphanou

**Affiliations:** Université Grenoble Alpes, CNRS, UMR 5525, VetAgro Sup, Grenoble INP, TIMC, 38000 Grenoble, France

**Keywords:** environmental stress, metabolic plasticity, systems biology, tumor metabolism

## Abstract

**Background**: The Warburg effect, historically regarded as a hallmark of cancer metabolism, is often interpreted as a universal metabolic feature of tumor cells. However, accumulating experimental evidence challenges this paradigm, revealing a more nuanced and context-dependent metabolic landscape. **Methods**: In this study, we present a hybrid multiscale model of tumor metabolism that integrates cellular and environmental dynamics to explore the emergence of metabolic phenotypes under varying conditions of stress. Our model combines a reduced yet mechanistically informed description of intracellular metabolism with an agent-based framework that captures spatial and temporal heterogeneity across tumor tissue. Each cell is represented as an autonomous agent whose behavior is shaped by local concentrations of key diffusive species—oxygen, glucose, lactate, and protons—and governed by internal metabolic states, gene expression levels, and environmental feedback. Building on our previous work, we extend existing metabolic models to include the reversible transport of lactate and the regulatory role of acidity in glycolytic flux. **Results**: Simulations under different environmental perturbations—such as oxygen oscillations, acidic shocks, and glucose deprivation—demonstrate that the Warburg effect is neither universal nor static. Instead, metabolic phenotypes emerge dynamically from the interplay between a cell’s history and its local microenvironment, without requiring genetic alterations. **Conclusions**: Our findings suggest that tumor metabolic behavior is better understood as a continuum of adaptive states shaped by thermodynamic and enzymatic constraints. This systems-level perspective offers new insights into metabolic plasticity and may inform therapeutic strategies targeting the tumor microenvironment rather than intrinsic cellular properties alone.

## Simple Summary

Tumor cells are often described as relying primarily on glycolysis, even in the presence of oxygen—a phenomenon known as the Warburg effect. However, recent evidence suggests that cancer metabolism is more flexible than this classical view implies. In this study, we developed a hybrid multiscale model that links cellular metabolism with the tumor microenvironment to explore how metabolic behaviors arise under changing conditions of oxygen, glucose, acidity, and lactate. Our simulations show that tumor cells can dynamically adapt their metabolic states depending on their local environment and stress history, without requiring genetic alterations. These results highlight that cancer metabolism should be seen as a spectrum of adaptive responses rather than a fixed trait, suggesting that targeting the tumor microenvironment could be an effective strategy to disrupt metabolic adaptation in cancer.

## 1. Introduction

This work investigates the adaptability of cellular metabolism in response to environmental disturbances encountered by tumors. One of the hallmarks of tumor metabolism, the Warburg effect—first described nearly a century ago—is often presented in contemporary literature as a universal feature of cancer cells. However, increasing experimental evidence indicates that this phenomenon does not consistently occur at the cellular level. In recent studies, we have highlighted the semantic drift associated with the Warburg effect, as well as with commonly linked concepts such as the *metabolic switch* and *metabolic reprogramming* [1,2]. While we do not overlook the critical role of mutations in tumor development, they are insufficient to fully account for the distinct features of tumor metabolism and are not necessary to explain many of the observed metabolic behaviors. A key message emerging from the literature is the necessity of integrating all biological scales to address the complexity of tumor behavior. This multiscale perspective is increasingly emphasized, supported by advances in instrumentation—particularly multi-omics approaches—and greater computational power. It remains essential to combine tissue-level and cellular-level measurements to capture the differences in dynamics [3]. If tumor metabolism can be understood as one possible state of a unified metabolic network, then it should be feasible to construct a model capable of generating these diverse metabolic states.

In this work, we develop a model of tumor metabolism that highlights spatiotemporal heterogeneity. This model enables the exploration of various environmental stress scenarios and their consequences on the metabolic trajectories of individual cells and the tissue as a whole. By combining continuous and discrete approaches within a hybrid multiscale framework, cell metabolism is spatially contextualized, allowing for the emergence of complex behaviors. The modeled environment includes four diffusive species: oxygen, glucose, lactate, and protons. Tumor tissue dynamics are represented using an agent-based model in which each cell acts as an individual agent characterized by properties such as metabolite concentrations, gene expression levels, cell cycle status, volume, and more—all of which are influenced by the local concentrations of environmental species through metabolite inflows and outflows. We recently developed a simplified model of cellular metabolism that emphasizes the role of acidity in glycolysis regulation and outlines the rules governing metabolic adaptation [4]. Our results suggested that what appears to be a cancer metabolic phenotype may not stem from intrinsic cellular abnormalities, but can instead emerge spontaneously in response to an overly acidic microenvironment [2]. Building on this earlier work, we propose a more comprehensive model of cell metabolism inspired by the framework of Li and Wang (2020) [5]. Their model is extended to incorporate the roles of lactate and acidity, including reversible lactate transport between the cell and its environment, the influence of acidity on transport direction, and the impact of acidity on glycolytic activity. These extensions are grounded in recent experimental findings [6,7]. This updated metabolic model was then integrated into the agent-based framework. The model’s behavior is tested under various environmental perturbations, including oxygen oscillations, acidic shocks, and glucose deprivation. This approach enables the exploration of more complex metabolic states—involving cooperative interactions between cells—and dynamic transitions across space and time.

The simulations presented here confirm our earlier findings, demonstrating that the Warburg effect is not ubiquitous and that temporal dynamics play a crucial role. The expression of this effect is not always directly correlated with a cell’s fermentative activity. On the contrary, the metabolic state of a tumor cell results from a subtle interplay between environmental conditions and internal cellular states shaped by the cell’s history. This state is not binary, but rather continuously adjusted through mechanisms available to the cell, enabling it to maintain homeostasis. The model suggests that these regulatory mechanisms are fundamentally thermodynamic in nature, mediated by enzymatic activity.

## 2. Model

### 2.1. A Hybrid Multiscale Framework

Tumor spheroids are widely used in vitro models for studying solid tumor growth due to their ability to reproduce key features of the tumor microenvironment, such as cellular heterogeneity and hypoxia—unlike conventional 2D monolayer cultures [8,9]. To investigate the heterogeneity of tumor metabolism under realistic spatial and temporal constraints, we developed a spatiotemporal model of a multicellular tumor mass, analogous to these in vitro spheroids. The model tracks thousands of individual cells, each with its own internal metabolic state, allowing the analysis of phenotypic evolution, spatial organization, energy profiles, and responses to environmental variations.

#### 2.1.1. Spatial Scales

**Figure 1:**
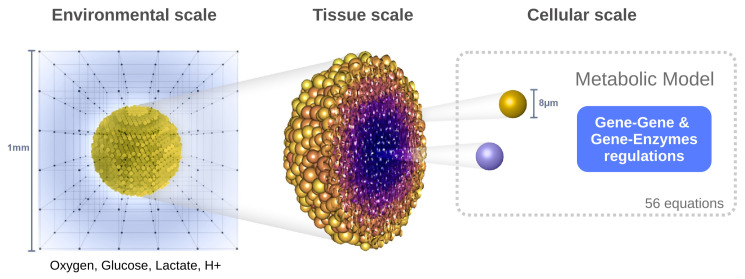
Structure of the hybrid multiscale framework: the simulation domain is defined by a grid in which the reaction–diffusion equations are solved for the environmental variables. The tumor spheroid is made up of interacting cell agents (tissue scale). The metabolic model is solved for each individual cells which possess their own characteristics and dynamics (cellular scale).

The model is structured within a hybrid multiscale framework encompassing three interconnected spatial levels (Figure 1):

- **Environmental scale:** The extracellular medium is described using reaction–diffusion equations for four key species: oxygen, glucose, lactate, and protons (to evaluate pH). This level captures the spatiotemporal distribution of diffusible molecules.
- **Tissue scale:** The tumor is modeled as a population of discrete agents, each representing a single cell. The agent-based model governs cellular behaviors such as movement, division, death, and mechanical interactions (e.g., adhesion, repulsion).
- **Cellular scale:** Each cell possesses its own dynamic metabolic model based on reaction kinetics (primarily Michaelis–Menten equations), which is locally influenced by environmental conditions. These intracellular dynamics determine the cell’s phenotype and fate over time.

This multiscale framework is implemented using the open-source software *PhysiCell* (version 1.7.1) [10], which supports 2D and 3D simulations. *PhysiCell* natively handles the numerical solution of partial differential equations (PDEs) for environmental variables using finite difference methods, and integrates standard cellular processes within the agent-based component.

#### 2.1.2. Temporal Scales

The modeled processes operate across different time scales, reflecting the natural disparity between fast metabolic reactions and slower cellular events. Assigning distinct temporal resolutions to each process allows the model to capture realistic dynamics while optimizing computational efficiency. The time steps used for each process are:

- **Diffusion:** 0.6 s
- **Metabolism:** 1.2 s (resolution of differential equations = 2× diffusion)
- **Mechanics** (e.g., movement, interaction): 6 s (10× diffusion)
- **Phenotype evaluation:** 6 min (600× diffusion)

### 2.2. Extracellular Environment

To investigate cellular energy metabolism, the model includes three primary substrates used by cells for ATP production: oxygen, glucose, and lactate. Additionally, protons (H+) are incorporated as a fourth environmental variable, due to their key role in metabolic regulation, as previously demonstrated in our reduced model of cellular energy metabolism [4]. The concentration ranges and boundary conditions used for each substrate are as follows:

- **Oxygen**: Extracellular oxygen varies significantly within tumor tissues. Peripheral cells consume oxygen rapidly, leading to depletion in the spheroid core. As oxygen diffuses passively into cells, intracellular concentrations are assumed to equilibrate with extracellular levels. Physiological values range from 160 mmHg in air to 70 mmHg in arteries and 38 mmHg in healthy tissues, and they drop below 15 mmHg under hypoxia, reaching pathological hypoxia below 8 mmHg [11]. In our simulations, initial oxygen concentrations range from 0 to 38 mmHg (converted to mM using the Valabrègue coefficient, $1.30\times 10^{-3}$ [12]). Dirichlet boundary conditions are applied to mimic continuous oxygen exposure, as in spheroid cultures. The diffusion coefficient is $87,600\;\mu m^{2}/\min$ [13].
- **Glucose**: Glucose is also consumed by cells and enters via facilitated diffusion through GLUT transporters. Its average blood concentration is approximately 5–6 mM. As the culture medium is regularly renewed in spheroid experiments, glucose concentration at the domain boundaries is held constant (Dirichlet condition). The diffusion coefficient used is $30,000\;\mu m^{2}/\min$ [14].
- **Lactate**: Lactate is primarily produced by cells and exchanged with the environment via monocarboxylate transporters (MCTs) in the form of lactic acid, co-transported with protons. At the domain boundaries, lactate concentration is generally fixed at zero (Dirichlet condition), reflecting regular renewal of the medium. In specific simulations, zero-flux (Neumann) conditions are applied to allow lactate accumulation. The diffusion coefficient is $12,600\;\mu m^{2}/\min$ [15].
- **H+**: Proton dynamics are coupled to lactate transport, entering and exiting the cell through MCTs. The resulting extracellular pH typically ranges from physiological (7.4) to acidic (4.0) values. A fixed boundary pH of 7.4 is imposed (Dirichlet condition), although in some simulations, zero-flux boundaries are used. The diffusion coefficient for protons is $270,000\;\mu m^{2}/\min$ [16].

### 2.3. Cell Metabolism

We recently developed a reduced model of cellular metabolism that emphasized the regulatory role of acidity [4]. The present, more detailed model retains the main hypotheses of that earlier work while extending its scope.

Our approach builds upon the model by Li and Wang (2020) [5], which itself refines the glycolysis-focused framework of Marín-Hernández et al. (2011) [17] by incorporating mitochondrial energy pathways, enzyme concentrations, and the regulation of ten key genes. The Li and Wang model couples a gene regulatory network, expressed as a system of ordinary differential equations (ODEs), with metabolic processes. Compared to our previous reduced model [4], it provides a finer biochemical resolution while maintaining a similar modeling philosophy. Parameters are drawn from experimental data, and the system is numerically stable. To capture intrinsic biological variability, stochastic terms are included, allowing the analysis of state distributions rather than single deterministic trajectories. The resulting stationary-state landscape, and its associated probabilities, can be mapped as in [5].

In the original implementation, the analysis focused on two variables—PDH (pyruvate dehydrogenase) and LDH (lactate dehydrogenase)—representing the bifurcation between respiration and fermentation. While insightful, this formulation did not address several questions central to our study, that we now integrate. Specifically:

- Temporal dynamics of individual cell states is tracked, to follow time-resolved trajectories.
- The extracellular environment is updated dynamically, and pH—which can reshape the metabolic landscape—is included.
- Spatial heterogeneity within a growing tumor mass is considered.

To that aim, we adapted the Li and Wang equations to explicitly include temporality, spatial context, lactate transport mechanisms, and the influence of pH. This extension allows the model to capture spatiotemporal metabolic heterogeneity and to integrate environmental feedbacks relevant to tumor growth. The model adaptations are described in the Appendix A section.

### 2.4. Cell Cycle and Cell Death

The core mechanisms governing the cell cycle and cell death are natively implemented in PhysiCell [10]. Below, we describe the specific adaptations introduced in our model.

#### 2.4.1. Cell Cycle

The model is calibrated on U87 human glioblastoma cells, whose cell cycle lasts approximately 18–24 h [18]. The first G1 phase has a baseline duration of 5 h. During G1, the cell evaluates two conditions at *checkpoint 1*: (i) whether the ATP level is sufficient to sustain progression, and (ii) whether the local cell density is below a threshold. If either condition fails, the cell enters the quiescent G0 phase (*checkpoint 2*), where it remains until conditions improve. Tumor cells can divide under higher densities than healthy cells, but a maximal tolerable density is still imposed, beyond which they enter G0. If oxygen levels decrease (without reaching hypoxia), the G1 phase duration is proportionally extended (*checkpoint 3*) to delay the irreversible transition to S phase in case of further deterioration [10]. Upon entering S phase, the cell doubles its volume (*checkpoint 4*) in preparation for division. At the end of G2, during the M phase, the γ parameter (described in the Appendix A section *—Regulation of gene expression*) is reassigned to introduce gradual epimutational variability. At mitosis completion, the cell divides into two daughters of equal volume, sharing identical parameters and concentrations, but evolving independently thereafter. While all cells share the same cycle structure, the population remains asynchronous: each cell’s initial phase is assigned randomly, weighted by phase durations.

#### 2.4.2. Cell Death

Cells may undergo necrosis or apoptosis. Although tumor cells evade apoptosis, they remain susceptible to necrotic death under severe energy depletion. In the model, cell death is triggered when ATP concentration drops below a critical threshold of 0.3 mM, consistent with reported death thresholds of 15–25% of basal ATP [19]. The probability of necrosis increases with the depth of ATP depletion. To avoid death from transient ATP dips (e.g., due to local competition for nutrients), the deficit must persist for at least 3 h. Necrosis proceeds in two stages: (i) necrotic swelling, where cell volume increases, followed by (ii) volume reduction until the cell disintegrates into debris. Upon lysis, glucose, oxygen, lactate, and intracellular protons are released into the extracellular space, where they diffuse freely.

## 3. Results

The simulations were designed to first establish a reference context, representing a typical in vitro spheroid growth experiment. This reference simulation was fully characterized to serve as a baseline for comparison with simulations performed under altered environmental conditions. We then introduced perturbations, including cyclic hypoxia, acid shock, and glucose deprivation. Finally, we challenged the glycolytic metabolism to examine whether, and how, cancer cells adapt to new constraints. The results are presented in the following three dedicated sections.

### 3.1. Reference Simulation

Spheroids are a valuable in vitro model for tumor growth [20] because (*i*) spatial heterogeneities arise naturally due to limited nutrient and oxygen diffusion from the periphery to the core, and (*ii*) environmental parameters such as oxygen or glucose concentrations can be readily manipulated. We therefore simulated a spheroid cultured in a round-bottom, low-attachment well.

#### 3.1.1. Initial Conditions

**Figure 2:**
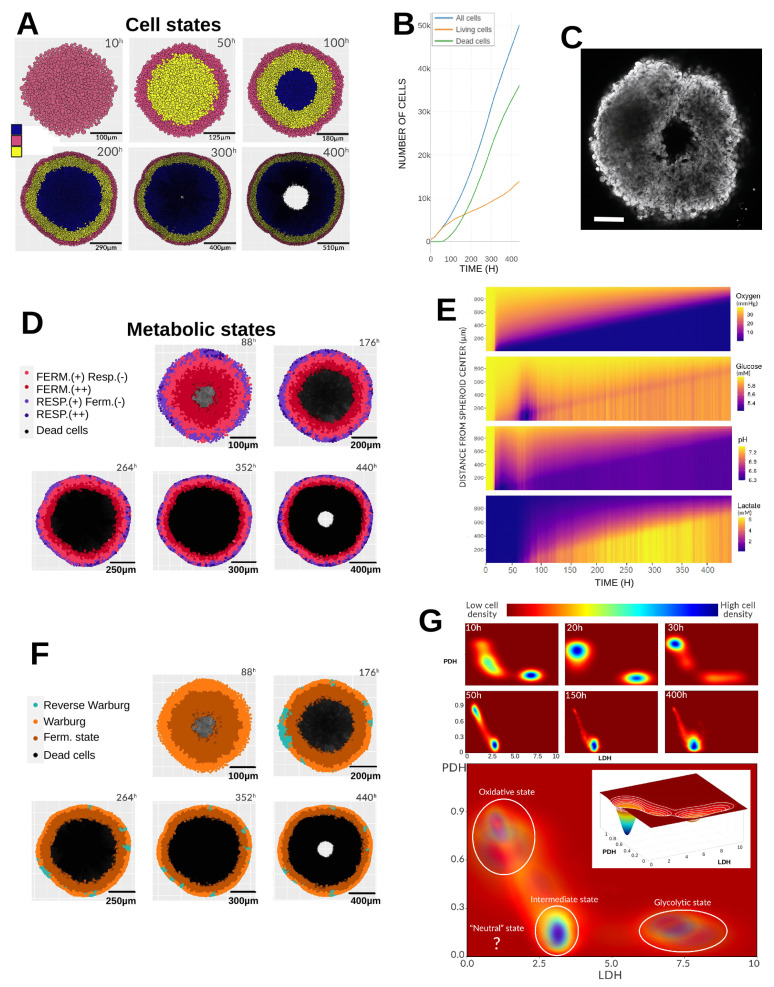
Reference simulation. (**A**) Temporal evolution of cell states in the spheroid from 10 to 400 h. Blue: necrotic cells; yellow: quiescent (G0) cells; pink: proliferative cells (G1, S, G2, M); black: cellular debris gradually cleared from the domain. (**B**) Dynamics of the different cell populations over time. (**C**) Illustrative image of an F98 (rat glioma) spheroid, diameter 390 μm. (**D**) Evolution of metabolic phenotypes. Cell sizes are shown uniformly for clarity. Symbols: “−” indicates low levels of fermentation/respiration; “+” indicates dominant metabolism. Black: necrotic cells. (**E**) Mean radial profiles of oxygen, glucose, lactate, and pH in the extracellular medium over time. (**F**) Occurrence of Warburg and reverse Warburg-type effects. Light orange: Warburg effect; dark orange: fermentation state; light blue: reverse Warburg-type effect (lactate uptake). Black: necrotic cells. (**G**) Temporal evolution of the metabolic landscape based on LDH and PDH gene expression. Color scale: red = low cell density, blue = high cell density. The time integration of cell metabolic states highlights the resulting metabolic landscape of the simulation which is shown below, with white circles marking the attractor regions (i.e., major density zones). Inset: distribution of cell densities at 30 h, with white lines indicating iso-density contours.

In the reference simulation, glucose was set to 6 mM at t=0, while oxygen was fixed at 38.0 mmHg, a physiologically relevant tissue-level pressure. Lactate was initialized at a minimal concentration (0.1 mM), and pH was set to its physiological value of 7.4. These concentrations, as well as pH, were maintained constant at the simulation domain boundaries (Dirichlet conditions), mimicking continuous renewal of the culture medium to sustain glucose availability, remove lactate, and stabilize pH via buffering. Oxygen was kept constant through passive exchange with the incubator atmosphere. Although the model supports full 3D simulations, we restricted this study to 2D due to computational cost. As a result, cells moved within a plane, and the “2D spheroid” diameter expanded faster than that of a real 3D spheroid. The objective was not to match spheroid size or growth rate quantitatively, but to identify common emergent structures and dynamics. Figure 2 illustrates the reference simulation with these initial and boundary conditions. At t=0 h, the spheroid contained 440 cells; by t=443 h, when the spheroid reached the boundaries of the simulation domain, it contained approximately 50,000 cells. Throughout growth, morphology remained homogeneous, but a necrotic core progressively developed. Dead cells were removed from the domain by *PhysiCell*, producing a central cavity closely resembling experimental observations (Figure 2C).

#### 3.1.2. Gradients and Radial Profiles

A key advantage of spheroids is their approximate radial symmetry, which simplifies characterization of emergent heterogeneity along radial profiles. This heterogeneity arises from spatial gradients of substrates that are consumed or produced at different rates from the spheroid periphery to its core. The first emergent pattern is the distribution of cellular states (Figure 2A). Initially, the spheroid consists solely of proliferating cells. By t=50 h, a thin quiescent layer appears, reflecting crowding-induced growth arrest and reduced nutrient availability. At t=100 h, a necrotic core emerges due to oxygen and glucose depletion and the accumulation of acidic metabolites. This core persists until the end of the simulation. After t=300 h, central necrotic debris is cleared, leaving a cavity (Figure 2C). Radial concentration profiles (Figure 2E) are obtained by averaging the concentration in all voxels equidistant from the spheroid center. During the first ∼20 h, oxygen and glucose diffusion outpaces consumption, preventing depletion. This interval corresponds to the time required for all cells to complete at least one cell cycle, increasing density and metabolic demand. Oxygen depletion then rises nearly linearly, with only peripheral cells having full oxygen access. Deeper cells experience partial oxygen supply, rapidly consuming any molecules that diffuse inward. pH decreases progressively, and this directly reflects glycolytic lactate production in the model since other mechanisms for acidity regulation are not taken into account. Between t=50 and 100 h, a transient pH normalization coincides with a sharp drop in glucose, indicating pyruvate shortage and compensatory lactate uptake, which also consumes protons. This temporarily relieves glycolytic inhibition, increasing glucose consumption before lactate availability declines again, re-acidifying the core. After t=100 h, lactate accumulates but cannot be oxidized due to persistent hypoxia, leaving only peripheral layers capable of oxidative metabolism. These layers consume both oxygen and glucose, producing lactate that further acidifies the inner core.

#### 3.1.3. Emerging Phenotypes

Figure 2D shows metabolic phenotypes based on pyruvate flux partitioning between fermentation (reaction r12, lactate dehydrogenase) and respiration (reaction r18, pyruvate dehydrogenase; see Table A1 in the Appendix A section). Cells are classified as predominantly fermentative if r12>r18, with pronounced fermentation defined as r12>10×r18 (i.e., more than 90% of the pyruvate is directed to lactate production). Analogously, r18>r12 defines respiratory cells, with pronounced respiration when r18>10×r12. Peripheral cells consistently exhibit oxidative metabolism (purple), often with some fermentation. Some transiently display pronounced respiration (dark purple). Below this layer, cells ferment more due to oxygen scarcity and lactate accumulation. The deepest viable cells ferment exclusively. Phenotypes can also be categorized according to the Warburg or reverse Warburg-type effects (Figure 2F).

The Warburg effect is defined as the net excretion of lactate by the cell, without applying any quantitative threshold to qualify this behavior. Similarly, the reverse Warburg-type effect corresponds to the net uptake of lactate. Thus, the direction of the lactate flux—outward versus inward—determines the metabolic status of the cell. We note that the *reverse Warburg effect* terminology was initially proposed by Pavlides et al. [21] to describe the metabolic symbiosis where stromal cells feed cancer cells with lactate. Here we refer to this terminology to denote a similar process occurring between tumor cells, where lactate produced by some cancer cells is utilized by others. Warburg-like behavior dominates, even in cells labeled as respiratory, though their lactate excretion is much lower than that of hypoxic cells. Importantly, lactate production does not always imply excretion, and vice versa, since intra- and extracellular lactate levels can equilibrate [6]. This raises questions about precise, operational definitions of Warburg and reverse Warburg-type phenotypes at the single-cell level.

#### 3.1.4. Metabolic Landscape

The metabolic landscape is defined using the expression levels of the LDH and PDH genes. These genes govern the fate of pyruvate (r12 vs. r18) and vary more slowly than metabolite levels, capturing medium-term environmental effects. They serve as respective markers of fermentative (glycolytic) and oxidative (respiratory) metabolism, following the approach proposed by Li and Wang (2020) [5]. Since the full gene metabolism network involves 53 variables, this high-dimensional system is projected onto a two-dimensional space spanned by LDH and PDH, capturing the balance between glycolysis and oxidative phosphorylation that characterizes the metabolic state of each cell. The metabolic landscape defines attraction basins corresponding to three states: oxidative cancer, glycolytic cancer, and intermediate cancer. Figure 2G shows that cells occupy three of the four states. Early in the simulation, they cluster into glycolytic (bottom right) and oxidative (top left) zones, matching the fermentation–respiration distribution in Figure 2D. At t≈30 h, medium acidification with residual oxygen drives a temporary oxidative shift, visible as a distinct excursion in Figure 2G (inset). However, by t=50 h, hypoxia forces cells toward a hybrid intermediatel state, which dominates thereafter. The boundary between oxidative peripheral cells and intermediate inner cells remains sharp until the simulation ends.

### 3.2. Environmental Perturbations

#### 3.2.1. Cyclic Hypoxia

Cancer cells are frequently subjected to cycles of hypoxia, largely due to the complex and dynamically reorganizing tumor vasculature [22]. Experimental studies have exposed tissues to intermittent oxygen tensions ranging from 10 to 60 mmHg (occasionally up to 160 mmHg, though such levels are not physiological) [23]. These oscillations can occur at fast (sub-hour), intermediate (tens of hours), or slow (multi-day) frequencies [22,23]. Understanding how such fluctuations influence cellular metabolism, and how rapidly cells can adapt, is of considerable interest. To explore this, we performed three simulations based on the reference configuration but imposed cyclic variations in oxygen at the domain boundaries: alternating between a high level (70 mmHg) and a hypoxic level (15 mmHg). These values were chosen slightly above the experimental range to account for the intratumoral depletion gradient. Cycles of 18, 36, and 72 h were applied, with each oxygenation or hypoxic phase lasting half the cycle period. All other intra- and extracellular parameters were identical to the reference simulation.

**Figure 3:**
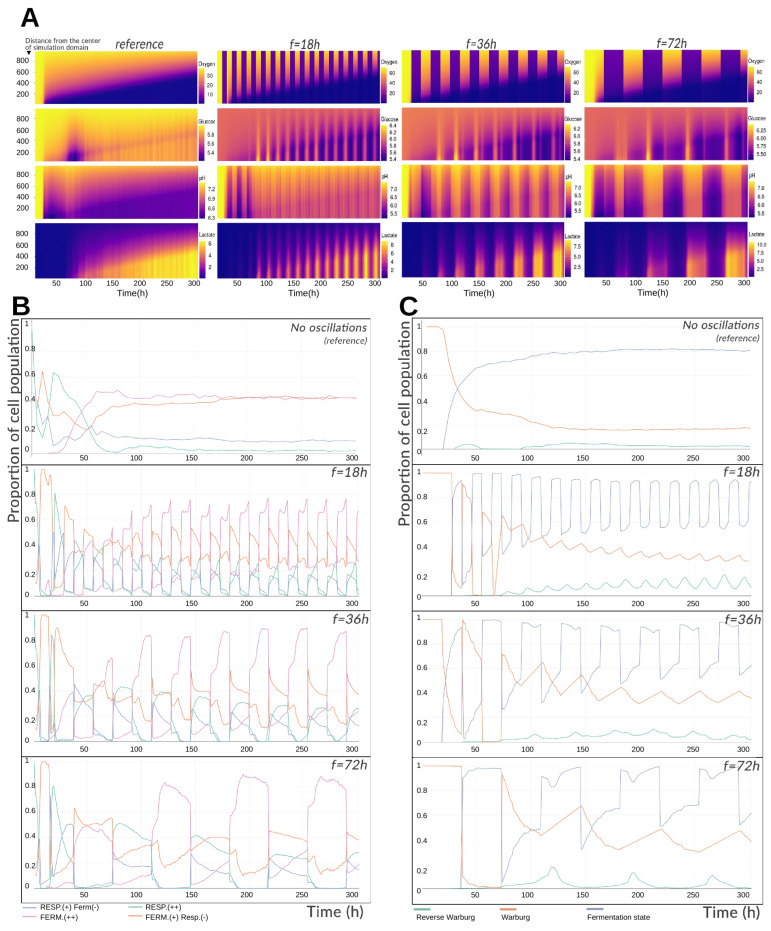
(**A**) Mean radial profiles of extracellular substrate concentrations and pH during imposed hypoxia cycles. Results are shown for three 300 h simulations in which oxygen at the boundary alternates between normoxic and hypoxic levels with periods of 18, 36, or 72 h. The ordinate indicates radial distance from the center of the simulation domain (in μm); color codes represent extracellular concentrations (mmHg for oxygen, mM for glucose and lactate, dimensionless for pH). (**B**) Temporal evolution of the proportion of metabolic phenotypes in the spheroid under cyclic or constant oxygenation. Phenotypes labeled with (−) indicate a lower relative contribution than those labeled with (+). (**C**) Temporal evolution of the fractions of cells exhibiting the Warburg effect or the reverse Warburg-type effect in the spheroid under cyclic or constant oxygenation.

Figure 3A shows that oxygen oscillations produce depletion fronts for oxygen and lactate that advance similarly to the reference case, indicating comparable spheroid growth. However, concentrations at these fronts differ: oxygen alternates sharply between imposed limits, while lactate accumulates at slightly higher levels (8–10 mM vs. 6 mM in the reference simulation), peaking during hypoxic phases. Lactate increases are synchronous with oxygen drops and persist throughout hypoxia. pH, however, behaves differently: although it also changes in phase with oxygen drops, it rises rather than falls during hypoxia—counter to expectations from fermentation-driven proton export. This discrepancy is explained by glucose profiles: about 6 h into hypoxia, glucose transiently spikes (sometimes exceeding boundary concentrations) before declining for the remainder of the phase. This spike corresponds to synchronous cell death events, releasing intracellular contents. Because intracellular pH is higher than the extracellular environment, these deaths cause a temporary extracellular alkalinization alongside lactate and glucose increases. Upon reoxygenation, pH and lactate decline.

Figure 3B depicts the proportion of each metabolic phenotype among living cells over time. Early in the simulation, cells converge from non-equilibrium initial conditions toward metabolic attractors, causing transient variations in both reference and oscillatory cases. Oxygen oscillations slightly delay but do not prevent stabilization, which occurs after ∼50 h. During hypoxia, fermentation dominates (75% for 18 h cycles, up to 90% for 72 h cycles). During oxygenation, the “Fermentation (+)/Respiration (−)” hybrid phenotype becomes predominant (40–55%). Across all scenarios, respiration declines over time due to increasing tissue size and oxygen demand. While phenotypes are defined discretely here, transitions are continuous—dominant fermentation states differ only slightly from dominant respiration states. Remarkably, phenotype distributions remain nearly constant from cycle to cycle, underscoring the short-term adaptability of tumor metabolism to oxygen fluctuations.

Warburg and reverse Warburg-type effect dynamics are shown in Figure 3C. In the reference simulation, ∼18% of cells express the Warburg effect, while ∼80% of cells excrete lactate under normoxia, yielding ∼98% total lactate-producing cells. Under oxygen oscillations, global trends remain, but local variations are substantial: lower oscillation frequencies produce larger amplitude shifts in Warburg expression, as longer cycles allow cells to converge fully to a metabolic attractor before switching.

In particular, 72 h cycles yield prolonged fermentation phases with higher lactate output, followed by sufficient reoxygenation time for the reverse Warburg-type effect to manifest and return to baseline. In contrast, 18 h cycles generate less lactate overall but show a slightly elevated baseline reverse Warburg-type activity, as frequent switching prevents complete equilibration of intra- and extracellular lactate. At peak, the reverse Warburg-type effect accounts for ∼19% of the tissue’s metabolic activity, comparable to the basal Warburg expression in the reference simulation.

#### 3.2.2. Acid Shock

We next investigated the metabolic consequences of a sudden decrease in extracellular pH, mimicking the acidic microenvironment encountered in tumors. In these simulations, the entire environment (not only the domain boundaries) was maintained at physiological pH (=7.4) for the first 50 h. From t=50 h onward, the pH was abruptly set to one of several constant values ≤7.4.

**Figure 4:**
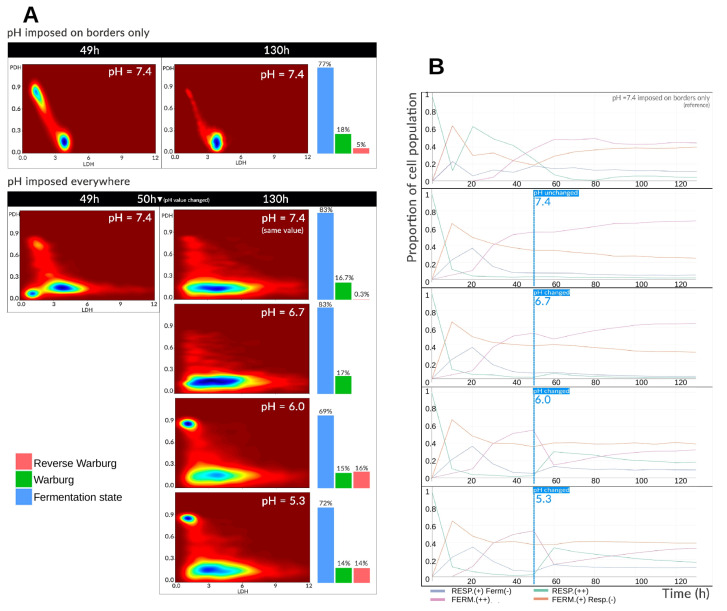
(**A**) Metabolic landscapes and Warburg-effect expression before and after a uniform pH shift applied to the entire environment. The “pH imposed only at the domain boundaries” condition corresponds to the reference simulation. Landscapes are shown at t=50 h (before the shift) and t=130 h (after the shift) for each pH condition tested. (**B**) Temporal evolution of the relative proportions of metabolic phenotypes in the spheroid population before and after the uniform pH change, compared to the reference simulation.

Figure 4A summarizes the results. The first panel recalls the reference simulation, in which pH is fixed only at the boundaries and allowed to evolve freely within the domain. The second panel presents the four pH conditions tested: 7.4 (physiological control), 6.7, 6.0, and 5.3. The metabolic landscape is shown both before (t=50 h) and after (t=130 h) the pH shift, allowing the metabolic landscape to converge to a stable configuration. Before the pH change, a marked difference is observed between the boundary-imposed case and the uniformly imposed pH case. When pH is imposed only at the boundaries, local acidification occurs at the spheroid core due to glucose consumption and lactate/proton excretion. This inhibits fermentation, concentrating cells in the *intermediate* zone of the landscape (LDH ≈ 4, PDH ≈ 0.1). A subset of oxygen-rich cells persists in the *oxidative* zone (PDH ≈ 0.9–1.0). In contrast, when pH is imposed uniformly, cells in the *intermediate* state occupy a broader region of the metabolic landscape, with distribution across all three attractors (*intermediate*, *oxidative*, *glycolytic*). The glycolytic attractor is least populated but still present. At t=130 h, the reference simulation shows most cells converging to the *intermediate* state as tissue growth progressively reduces oxygen availability. This pattern is also observed under fixed pH, but with a wider *intermediate* zone. Glycolytic states are more accessible when acidity is milder (pH=6.7–7.4) or has been imposed for a shorter time. At stronger acidities (pH≤6.0), a pronounced oxidative cell cluster reappears, consistent with glycolysis inhibition and sufficient lactate accumulation to induce the reverse Warburg-type effect. In these cases, the proportion of reverse Warburg-type cells approaches ∼15%, comparable to the fraction of Warburg-effect cells. Overall, pH exerts two distinct influences: (1) When uniform and neutral, it allows cells to explore a wider and more permissive metabolic space, with access primarily dictated by oxygen and glucose availability; (2) When sufficiently acidic, it inhibits glycolysis, whether through negative feedback from self-generated acidity or direct imposition by the environment.

Figure 4B illustrates the impact on metabolic phenotypes. At pH 7.4, fermentation is maintained more readily than in the reference simulation, as acidity does not inhibit glycolysis. Before t=50 h, phenotype distributions are identical across conditions. After the pH shift, differences emerge, most notably for pH≤6.0. At pH 6.7, only a slight transient drop in fermentative cells is observed, returning later to the pH 7.4 level, with reciprocal changes in respiratory metabolism. At pH 6.0 and 5.3, respiration and the “Respiration (+) Fermentation (−)” hybrid phenotype increase sharply, with respiration rates nearly matching those of the “Fermentation (+) Respiration (−)” phenotype by t≈60 h. This shift is accompanied by a ∼35% drop in purely fermentative cells. In the following hours, phenotype distributions partially reverse: fermentation increases and respiration decreases, but neither returns to physiological-pH levels. This rebound is driven by the progressive reduction in oxygen available to cells in the inner spheroid layers. With glucose in excess relative to oxygen supply, and given the capacity of cells to store glucose, fermentation resumes even under acidic conditions, albeit at a reduced rate.

#### 3.2.3. Glucose Depletion

In analogy with the pH perturbation experiments, we next investigated the impact of glucose deprivation on tumor metabolism. In our model, apart from oxygen, glucose (and lactate) are the only extracellular sources for replenishing pyruvate or acetyl-CoA in the absence of fatty acids or glutamine. This simulation therefore examines how tumor metabolism adapts when deprived of its principal carbon source.

**Figure 5:**
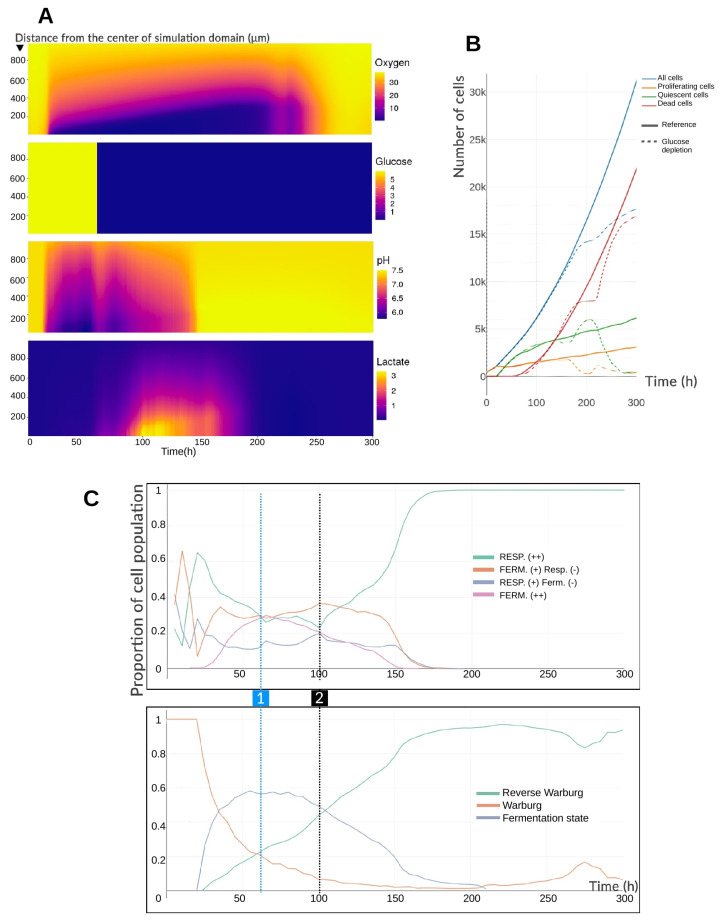
(**A**) Mean radial profiles of extracellular substrate concentrations before and after glucose depletion at t=60 h. The ordinate represents the radial distance from the center of the simulation domain, and the color scale indicates extracellular concentrations (mmHg for oxygen, mM for glucose and lactate, and dimensionless for pH). (**B**) Temporal evolution of cell states in the spheroid population before and after the glucose drop in the entire environment. The glucose-depletion simulation (dashed lines) is compared to the reference simulation (solid lines). Glucose depletion begins at t=60 h. (**C**) Evolution of the proportions of metabolic phenotypes and the expression of the Warburg effect in the cell population over time, before and after glucose depletion. (1) At t=60 h, extracellular glucose is fixed at 0.01 mM throughout the environment. (2) At t=100 h, intracellular glucose levels have equilibrated with the depleted extracellular concentration. Phenotypes labeled with (−) indicate a lower relative contribution than those labeled with (+).

The spheroid initially grows under the same conditions as in the reference simulation, except that the glucose concentration is fixed at 6.0 mM throughout the environment (not only at the domain boundaries). Because the glucose gradient is negligible before t=60 h in the reference case, it is possible to maintain this uniform value without altering early dynamics. At t=60 h, the glucose concentration is abruptly reduced to 0.01 mM throughout the environment and held constant thereafter. This drop is shown in Figure 5A. The simulation is run for 300 h in total—longer than in the pH experiments—because the metabolic consequences of glucose depletion emerge more slowly. Unlike in the previous perturbations, here the dynamics of oxygen, pH, and lactate are not monotonic. The acidic pH gradually returns to physiological levels by ∼150 h. Lactate peaks at ∼3 mM around 100 h, then declines to nearly zero by 200 h. Oxygen depletion proceeds similarly to the reference case until ∼230 h, after which a brief rebound is observed (230–240 h), followed by a transient depletion episode, and finally a sustained return to the initial concentration of 38 mmHg.

Figure 5B clarifies these dynamics. Cell numbers and cell-state distributions remain close to the reference until ∼150 h, when the number of proliferating and quiescent cells begins to decline. Proliferating cells continue to decrease until ∼200 h, while quiescent cells initially increase before rapidly falling after 230 h. The number of dead cells temporarily stabilizes, then rises sharply around 230 h. The brief oxygen burst between 230 and 240 h coincides with this loss of quiescent cells and a transient, small rise in proliferating cells. By the end of the simulation, only about a hundred cells remain viable. From a growth perspective, the spheroid shows little disturbance immediately after extracellular glucose depletion, with a delay of roughly 80 h before noticeable effects. This latency arises from the difference between extracellular and intracellular depletion kinetics. Whereas intracellular oxygen equilibrates rapidly via passive diffusion, intracellular glucose is imported by facilitated diffusion [24], which—although faster than simple diffusion—depends on the abundance of GLUT transporters. During this lag phase, cells continue to consume their internal glucose stores, even as the extracellular concentration is kept constant at its depleted value. Once intracellular glucose levels drop, pyruvate production from glycolysis declines. To sustain ATP production, cells begin consuming lactate, converting it into pyruvate. This lactate depletion results both from intracellular consumption and from the exhaustion of extracellular lactate. By ∼200 h, nearly all extracellular lactate has been taken up, raising extracellular pH, and from ∼210 h onward, most cells have exhausted their intracellular lactate. With no pyruvate supply, respiration ceases, oxygen accumulates, and ATP production becomes insufficient. Most cells die, while a few survivors benefit from ideal oxygenation, residual glucose, and the release of glucose and lactate from lysed neighbors.

Figure 5C confirms the delayed metabolic response. Before 60 h, the distribution of phenotypes and Warburg-effect expression is similar to the reference case (Figure 3C), though with a slightly higher reverse Warburg-type effect due to faster intracellular lactate accumulation (no gradient). After 60 h, phenotypic proportions change little initially, except for the gradual decline in highly fermentative cells (“Fermentation (++)”) and continued rise of the reverse Warburg-type effect. From ∼100 h, when intracellular glucose equilibrates with the depleted extracellular level, respiration slows, then increases to become the dominant metabolic mode by ∼180 h. Notably, the Warburg effect persists longer than fermentation, as intracellular lactate levels continue to equilibrate with the environment and may still be transiently excreted. This illustrates that the Warburg effect does not always require concurrent high fermentation activity.

### 3.3. Challenging the Glycolytic Metabolism

The intrinsic heterogeneity of cellular metabolism makes it difficult to establish a universal law describing tissue evolution under varying environmental conditions. In practice, tissue-scale dynamics often dominate over the metabolic behavior of individual cells. Consequently, emergent behaviors in simulations are not always straightforward to predict. Nonetheless, from a large set of simulations, several recurring trends can be summarized (Table 1).

In general, lactate is excreted when oxygen levels are low or when lactate is already abundant in the environment. The latter condition increases HIF-1α expression, thereby stimulating glycolysis even in the presence of oxygen. Conversely, when lactate is absent and both glucose and oxygen are sufficient, respiration dominates and lactate production remains low (though never entirely absent). When extracellular lactate is abundant, cells tend to import it following the concentration gradient. This import can occur simultaneously with intracellular lactate production via fermentation, leading to accumulation inside the cell. Lactate uptake also occurs independently of its bulk concentration when oxygen is present but glucose is scarce, as it can serve to replenish pyruvate for mitochondrial respiration. Identifying these patterns is complicated by local variability in extracellular conditions. Fixing oxygen, glucose, lactate, or pH at the domain boundaries does not prevent localized microenvironments from forming within the spheroid. For instance, hypoxic cells in the core may produce large amounts of lactate, acidifying the periphery and forcing peripheral cells to metabolize lactate instead of glucose. Such cell–cell interactions are a major source of metabolic heterogeneity, often masked in experimental measurements that report only population-averaged behaviors [25].

To illustrate this, we consider a new simulation in which oxygen is fixed at atmospheric pressure (160 mmHg, in strong excess) while glucose is kept at only 1 mM, a limiting concentration. Lactate is initially absent, and the initial pH is acidic (6.7). Boundary conditions for lactate and protons are of Neumann type (zero flux), allowing them to accumulate in the domain over time. Cells thus have very limited glucose availability, and the acidic pH further suppresses glycolysis. The aim here is to test whether tumor cells maintain a glycolytic phenotype under conditions unfavorable to it—i.e., whether the Warburg effect is inevitable. Previous simulations suggested it is not, but this case also provides insight into emergent tissue-scale metabolic interactions.

**Figure 6:**
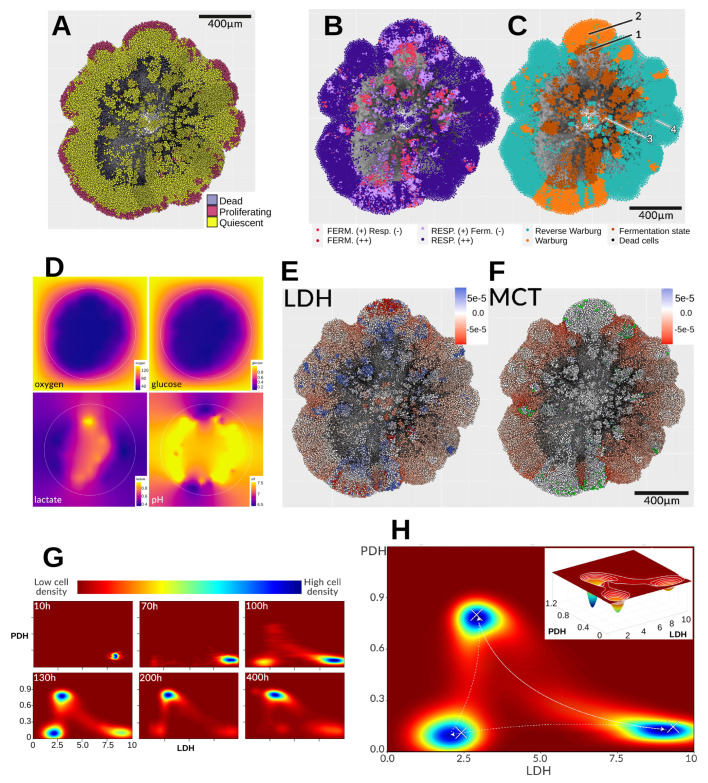
At 420 h, the spheroid exhibits pronounced spatial and metabolic heterogeneity. (**A**) Cell states reveal proliferative, quiescent, and necrotic (black) regions. (**B**) Dominant fermentation and/or respiration phenotypes, where “(–)” denotes lower prevalence than “(+)”. (**C**) Metabolic status: (1) fermentation state, (2) Warburg state, (3) dead cells, (4) Reverse Warburg-type state. (**D**) Extracellular gradients of oxygen (0–160 mmHg), glucose (0.2–1.0 mM), lactate (0.4–0.97 mM), and pH (6.49–7.49). (**E**) Flux analysis of LDH-catalyzed conversion (*r12*) and (**F**) lactate transport via MCT channels (*r21*); negative values indicate reverse flux (lactate→pyruvate or extracellular→intracellular transport). A small subset of high-producer cells (green in (**F**)) releases ∼20× more lactate than the rest; colors were chosen to avoid interference with the main flux scale. (**G**) Metabolic landscape evolution in LDH/PDH expression space: blue zones denote high cell density, red zones low density. (**H**) Time integration of cell metabolic states reveals attractor regions (crosses) in the simulated landscape, with the inset (130 h) showing the density distribution along the LDH/PDH bifurcation.

Figure 6A exhibits the cell states. As shown in Figure 6B, most cells preferentially oxidize pyruvate via respiration (dark purple). In hypoxic central regions, some clusters are forced to metabolize the small amount of glucose available. Many cells display a reverse Warburg-type phenotype, while a vertical, central cluster produces lactate despite high oxygen availability. Glucose, though scarce, is continuously supplied from the edges, and lactate accumulates over time (Figure 6D). Eventually, cell death (notably near zone 3 in Figure 6C) releases lactate, glucose, and protons into the surrounding tissue, where they are rapidly imported by neighboring cells. This creates an internal metabolic asymmetry and alters substrate diffusion patterns. Hypoxic cells (zone 1) convert their limited glucose into lactate and acid, which accumulate both intracellularly and extracellularly. This supports pyruvate regeneration and helps sustain ATP levels. Surrounding acidic environments cause zone 2 cells to reduce glucose use and release lactate (originating from zone 1) into the medium. The lactate then diffuses outward and is taken up by peripheral cells (zone 4), lowering the extracellular pH there. Thus, despite predominantly oxidative metabolism, peripheral cells actively import lactate as an alternative energy source, driving tissue-scale metabolic fluxes. Figure 6E,F confirm these processes: LDH flux maps show hypoxic cells in zone 1 converting pyruvate to lactate (blue), while peripheral cells in red perform the reverse conversion. MCT flux maps indicate that lactate import dominates at the periphery. Zone 2 cells appear mostly white, indicating low lactate export relative to import. A minority of highly glycolytic cells (green) export lactate at rates ∼20-fold higher (∼10^−3^ mM/min) than others.

These results reinforce that the Warburg effect is not universally present at the single-cell level, even when it may be apparent macroscopically at the tissue scale. As discussed previously [1,2], current definitions of the Warburg effect are insufficient to capture precise metabolic behavior at the cellular scale, let alone to define threshold criteria (e.g., “production of *X* lactate in the presence of *Y* oxygen”). Even by the original Otto Warburg definition—lactate production regardless of oxygen levels—this behavior is not ubiquitous. From a metabolic landscape perspective, three of the four possible attractor zones emerge (Figure 6G). Initially (∼10 h), cells exploit the limited glucose by adopting a *glycolytic* phenotype (bottom right: low PDH, high LDH). By ∼70 h, acidification drives some cells toward a less glycolytic phenotype (bottom left), moving along the LDH axis without major PDH changes. By 100 h, persistent glucose depletion forces glycolytic and intermediate cells toward the *oxidative* phenotype (top left). Glycolytic cells in particular transition directly to the oxidative state without passing through the intermediate zone—a shift especially visible after 130 h (Figure 6H) and persisting until the end. At 400 h, the population is largely divided between oxidative and glycolytic states. However, this is an aggregate view: although most cells cluster around these metabolic attractors, transitions between states continue throughout the simulation, as shown by the connecting trajectories in the attractor space. The final panel of Figure 6H depicts the aggregation of these three attractors and the dynamic trajectories linking them.

## 4. Discussion

The importance of hybrid multiscale models of multicellular spheroids for investigating tumor metabolism has been emphasized in recent reviews [26,27]. Our work follows this line of development. The originality of our approach lies in challenging the classical paradigm that attributes tumor metabolic diversity primarily to genetic alterations. We demonstrate instead that a wide range of metabolic phenotypes can emerge without mutations, purely as a result of dynamic interactions between cells and their microenvironment. This is made possible by the metabolic model we use, adapted from that of Li and Wang (2020) [5], which incorporates the regulatory influence of gene networks on metabolic behavior.

By embedding a mechanistically grounded metabolic network within an agent-based spatial framework, our multiscale model explicitly couples intracellular metabolic adaptation to microenvironmental dynamics. This design captures the evolving metabolic phenotypes driven by environmental fluctuations accompanying tumor growth—this highly dynamical aspect is generally missing from omics-based approaches [28]. Compared with single-cell studies, which provides static snapshots or metabolomics studies which are most often based on population-level, our model offers a causal and predictive framework to explore how local stresses (oxygen oscillations, acidity, nutrient deprivation) shape emergent metabolic heterogeneity over time. The added value of the model is its ability to resolve both spatial and temporal dimensions of tumor metabolic adaptation.

High-throughput multi-omics studies (e.g., refs. [28,29,30]) provide broad empirical snapshots of tumor metabolic states and identify associations between gene, protein, and metabolite signatures and clinical or phenotypic variables. While these studies offer patient relevance and molecular correlates of metabolic phenotypes, they are limited by snapshot sampling, measurement noise, and difficulty inferring causal or environment-dependent dynamics from steady-state data. By contrast, our hybrid multiscale model is mechanistic and dynamic: it represents thermodynamic and enzymatic constraints, reversible lactate transport, and microenvironmental gradients, allowing simulation of spatiotemporal adaptation to perturbations (e.g., oxygen oscillations, acidic shocks). Although simplified, it provides interpretable causal hypotheses and testable predictions about how microenvironmental changes shape metabolic phenotypes. Together, empirical multi-omics and mechanistic modeling are complementary: omics can inform and constrain models, and models can generate mechanistic interpretations and guide targeted experiments.

The simulations carried out made it possible to identify in an emergent way, different metabolic behaviors which are spatiotemporally defined. The reference simulation showed the standard radial distribution of cell states (proliferative, quiescent, and necrotic) within a spheroid. It showed the impact of the underlying metabolism on the environment with mainly a strong oxygen depletion in the internal layers of the spheroid and an increase in lactic acidosis. These profiles correspond to classical observations of spheroids in vitro [31,32]. It also highlighted a partial decoupling between the Warburg effect and the type of metabolic phenotype. Essentially respiratory cells can express the Warburg effect by secreting lactate. This results in a marked heterogeneity of the distribution of the cells of the spheroid within the metabolic landscape defined by the expression of the LDH and PDH genes. Ultimately, however, an intermediate metabolic state predominates in the tissue.

Simulations of intermittent oxygen concentration have shown the short-term stability of metabolism to these variations as well as the resurgence of the reverse Warburg-type effect on tissue reoxygenation phases. The acid shock simulations also showed the stability of the metabolism in the face of sudden pH variations. More acidic pH allows the reverse Warburg-type effect to be more strongly expressed (phenomenon experimentally observed [6]) which is also reflected in the metabolic landscape by an increase in the expression of PDH. Depletion of extracellular glucose has a slower effect, depending on intracellular reserves. The metabolic impact is limited as long as lactate (and by extension other sources to continue to fuel respiration) is available. Once these sources are exhausted, this condition is most critical for the survival of rapidly dying cells.

The simulations highlight the complexity of the multiple behaviors that can take place within the same tissue, with cyclic hypoxia phenomena when the external conditions are at the limit barely sufficient to allow a few cells to proliferate. There can also be phenomena of indirect cellular collaborations, of “metabolic symbiosis” [33], the metabolic products of a cell exposed locally to certain environmental conditions benefiting another cell exposed to different conditions.

As a consequence, it is possible to conclude that the metabolism is relatively robust in the face of environmental disturbances, at least over short periods of time (<1 month). In reality, what introduces instability in the metabolic organization of a tissue does not come intrinsically from the metabolism, but from the existence of abnormal conditions in which the cells are pushed (the depletion of glucose in the environment resulting in death is an example). A spheroid, too, is an abnormal tissue configuration due to its high density or the absence of vascularization. The metabolism itself has all the biochemical and thermodynamic properties [34] to continue to be operational from the point of view of maintaining cell viability. Targeting tumor metabolism will require strategies that account for its spatial, temporal, and cooperative plasticity—attacking not just the cell, but the community it sustains.

The insights provided by this model have several therapeutic implications. First, the emergence of both the Warburg and reverse Warburg-type effects under different microenvironmental stresses supports the idea that targeting metabolic fluxes rather than static phenotypes may be more effective. In particular, inhibiting lactate shuttling between cells, for instance through monocarboxylate transporter (MCT1/MCT4) inhibition, could disrupt metabolic cooperation and limit tumor growth. Likewise, interventions aiming to regulate extracellular pH—either by buffering tumor acidity or modulating proton transporters—could stabilize the metabolic equilibrium. Our model suggests that such strategies may be most effective when applied dynamically, in synchrony with transient environmental fluctuations (e.g., oxygen cycles), to prevent metabolic re-adaptation.

The current model focuses on glucose and lactate metabolism; extensions could incorporate alternative nutrient sources such as fatty acids and glutamine, which are increasingly recognized as key fuels in cancer progression. Integrating vascularization dynamics would allow the study of perfusion heterogeneity, nutrient delivery, and therapy-induced reoxygenation effects. Coupling this framework with models of cell migration and extracellular matrix remodeling could further enable the exploration of invasion and metastasis as emergent consequences of metabolic and environmental adaptation. Ultimately, combining the present mechanistic model with experimental multi-omics or imaging data will provide a powerful tool for predicting tumor responses to metabolic or microenvironmental interventions.

## 5. Conclusions

The “Warburg effect” has long been used to describe the observation that tumor cells often engage in aerobic glycolysis, consuming large amounts of glucose and secreting lactate even in the presence of oxygen. However, both simulation-based and experimental studies indicate that this term can obscure the underlying plasticity and heterogeneity of tumor metabolism. In previous works, we highlighted that the widespread use of “metabolic reprogramming” and adherence to the classical Warburg paradigm often overlook dynamic variability of metabolic states [1,2].

Our multiscale simulations demonstrate that tumor metabolic behavior is not fixed around a single glycolytic attractor, but emerges dynamically from interactions between intracellular regulation and microenvironmental constraints. Cells may express a spectrum of metabolic phenotypes depending on local oxygen, glucose, and pH conditions. Notably, the Warburg phenotype is neither universal (present in all cells) nor constant (persistent over time). Under acidosis or lactate accumulation, the model predicts a shift toward a reverse Warburg-type state, characterized by lactate uptake and increased oxidative phosphorylation. These findings support the experimental view that tumor metabolism is better represented as a continuous landscape rather than a binary “glycolysis versus respiration” switch [1].

This broader perspective has practical implications. Considering tumor metabolism in terms of plasticity and heterogeneity, rather than a fixed Warburg state, suggests therapeutic strategies targeting dynamic metabolic fluxes under specific microenvironmental conditions. It also highlights the importance of spatial heterogeneity, temporal evolution, and metabolic coupling between cells, such as lactate shuttling, in shaping tumor behavior.

In conclusion, combining mechanistic multiscale simulations with experimental data enables a more nuanced understanding of tumor metabolism: not simply “Warburg or not,” but “where, when, and why” within the metabolic landscape. Recognizing and modeling this plasticity provides a foundation for more precise predictions, better experimental stratification, and rational metabolic targeting in cancer therapy.

## Figures and Tables

**Table 1:** General trends of simulations as a function of external conditions. The oxygen, glucose and lactate concentrations are reported qualitatively and the results column indicates the consequences of these conditions on the exposed cells (‘−’, ‘+’ and ‘++’ represent low, high and very high concentrations respectively).

Oxygen	Glucose	Lactate	Outcome
+	+	++	Lactate import
+	+	+	Lactate excretion
+	+	−	Low lactate excretion
+	−	+	Lactate import
+	−	−	Death
−	+	+	Lactate excretion
−	−	+	Death
−	−	−	Death

**Figure A1:**
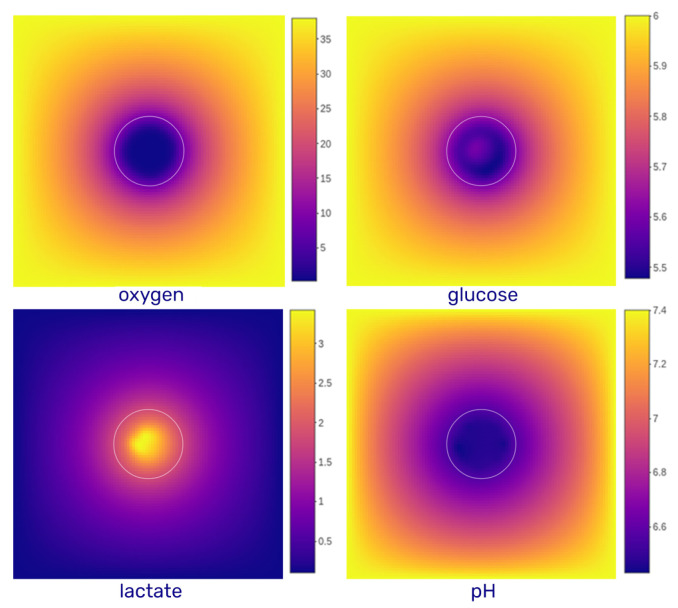
Diffusion profile for the environmental variables. The diffusion domain is a grid of 2000 μm in size (divided into 100 elements of 20 μm). The white circles represent the contour of the spheroid. The color gradient represents the concentration of each substrate. Oxygen is expressed in mmHg, glucose and lactate are expressed in mM and pH is dimensionless (pH is calculated from the proton concentration expressed in mM). Constant values are maintained at the domain boundaries for all variables (Dirichlet’s conditions).

**Figure A2:**
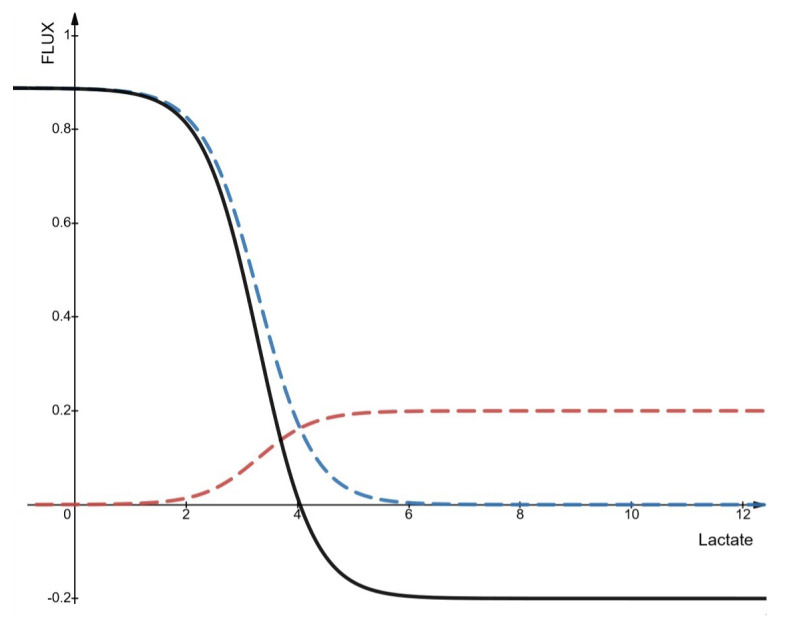
Incoming and outgoing flux of lactate and protons according to the extracellular concentration of lactate. In blue, the outgoing flux, in red the incoming flux (absolute values) and in black the resulting flux (r21). In this figure the concentration of pyruvate is 0.11 mM, intracellular lactate at 3.3 mM, the extracellular pH at 6 and the intracellular pH at 6.7 and $V_{mf_{21}}$ = 1.

## Data Availability

The code is made publicly available on GitLab.

## Supplementary Materials

The following supporting information can be downloaded at: https://www.mdpi.com/article/10.3390/cancers17213563/s1, File S1: Equations of the model of cell metabolism [5].

## Abbreviations

The following abbreviations are used in this manuscript:

ATP	Adenosine triphosphate
HIF	Hypoxia-inducible factor
LDH	Lactate dehydrogenase
MCT	Monocarboxylate transporter
PDH	Pyruvate dehydrogenase

## Appendix A. Model Details

### Appendix A.1. Non-Homogeneous Diffusion

Non-homogeneous diffusion is introduced in *Physicell* (version 1.7.1) by adding a dependency with regard to the local cell density ρ to better reflect the gradient’s intensity. The non-homogeneous diffusion is expressed as:

(A1) $$\frac{\partial C}{\partial t}=\nabla \cdot \left(D\left(\rho \right)\nabla C\right)$$

where *C* is the diffusive species. For each voxel, the cell density is evaluated (in % cell occupancy of the sampled volume). If the local density is 1 (100% of the surrounding voxel volume is occupied), then:

(A2) $$D\left(\rho \right)=D_{0}\cdot \left(1-\alpha \rho \right)$$

with $D_{0}$ the diffusion coefficient of the species in the interstitial liquid. α∈[0,1] is a parameter for adjusting the impact of the density on the diffusion, and ρ is the local density of the cells in the voxel. By using α = 0.25, the diffusion profiles obtained for the three substrates and pH (Figure A1) resemble those found in the literature [35].

### Appendix A.2. Model of Cell Metabolism

The biochemical model of metabolism takes up and modifies that of Li and Wang [5]. The differential equations describe the one- or two-way reaction flows and take the following form [36] which is an extended form of a Michaelis–Menten equation, reversible and extensible to several substrates [S] or several products [P]:

(A3) $$v_{net}=\frac{V_{maxf}\cdot \frac{\left[S\right]}{K_{ms}}-V_{maxr}\cdot \frac{\left[P\right]}{K_{mp}}}{1+\frac{\left[S\right]}{K_{ms}}+\frac{\left[P\right]}{K_{mp}}}$$

where $V_{maxf}$ and $V_{maxr}$ are, respectively, the maximum speeds of the reaction in the direct (forward) and reverse (backward) directions. $K_{ms}$ and $K_{mp}$ are the Michaelis constants of the substrate and the product, respectively.

Each cell in the simulation integrates the entire system of equations corresponding to its metabolism with its own parameters and concentrations. The cells are initiated with slightly different internal concentrations for each metabolite (around the values in the literature). At each time step ($dt_{ODE}$ = 1.2 s), cells are updated in a random order by solving the ODE system for each with the Rosenbrock solver of order 4, from the C++ Boost Odeint library [37], integrated in *PhysiCell*.

All equations are available in Supporting Information (File S1). Table A1 recapitulates the metabolic reactions of the model and highlights those that have been modified compared to the model of Li and Wang [5].

**Table A1:** Metabolic reactions integrated into the model. Green reactions have undergone minor changes compared to the original model, and major modifications are in orange.

IDs	Enzymes	Reactions
r1	GluT1	Glu_*out*_ ⇌ Glu_*in*_
r2	HK	Glu_*in*_ + ATP ⇌ G6P + ADP
r3	GPI	G6P ⇌ F6P
r4	PFK-1	F6P + ATP ⇌ FBP + ADP
r5	ALD	FBP ⇌ DHAP + G3P
r6	TPI	DHAP ⇌ G3P
r7	GAPDH	G3P + NAD+ ⇌ 1.3BPG + NADH
r8	PGK	1.3BPG + ADP ⇌ 3PG + ATP
r9	PGAM	3PG ⇌ 2PG
r10	ENO	2PG ⇌ PEP
r11	PKM2	PEP + ADP ⇌ Pyruvate + ATP
r12	LDH	Pyruvate + NADH ⇌ Lactate + NAD+
r13	G6PD	6PGD G6P ⇌ R5P
r14	ATPases	ATP ⟶ ADP
r15	AK	AMP + ATP ⇌2ADP
r16	PFKFB2	3F6P ⇌ F2.6BP
r17	PHGDH	3PG ⟶ Serine
r18	PDH	Pyruvate + ADP ⟶ Citrate + ATP + Complex2
r19	ACC	Complex2 + 3ATP + AC-CoA ⟶ mal-CoA + 3ADP + NAD+
r20	SOD	ROS ⟶ Null
r21		Lactate + $H^{+}$ ⇌ Lactate_*extra*_ + $H^{+}$
r22		3R5P ⇌ 2F6P + G3P
r23	Nucleotide Biosynthesis	R5P ⟶ Null
r24	Serine Consumption	Serine ⟶ Null
r25	GPDH	NADH + ADP ⟶ Complex2 + ATP + NAD+
r26		Citrate + 3ADP ⟶ 3ATP + 4Complex2
r27		Complex2 + 1.5ADP ⟶ 1.5ATP
r28		Complex2 ⟶ ROS
r29	NOX	null ⟶ ROS
r30		Citrate ⟶ Null

(MCT) For most of the reactions associated with the fermentation process (in green in Table A1), the expressions have been multiplied by a term $pH_{inhibition}$, which decreases the activity of the enzymes when the intracellular pH becomes acidic. The enzymes reported to be affected by the pH in the study by Xie et al. [6] are HK, GPI, PFK-1, ALD, TPI, GAPDH, ENO, LDH, G6PD-6PGD, PFKB2/3.

Inhibition term by pH

(A4) $$pH_{inhibition}=\alpha +\frac{1-\alpha }{1+e^{\left(\frac{pH_{mid}-pH_{e}}{\beta }\right)}}$$

with α, the minimum level of enzymatic activity at acidic intracellular pH (set from [6] at 0.1 = 10%), β defines the regulation sensitivity (set at 0.12) and $pH_{mid}$ is the pH at which the enzyme operates at 50% of its maximum activity (fixed at 6.7).

This term allows us to represent recent observations of an acidic-related reduction in the Warburg effect. This provides a source of heterogeneity within the tumor, particularly with respect to the gradients of exposure to acidity.

In the original model, oxygen concentration is treated as a fixed parameter with no direct effect on OXPHOS. Hypoxia increases HIF production, triggering downstream responses, while the respiratory chain is assumed to function normally, provided enough complex II units are formed. In reality, oxygen acts as the final electron acceptor. Therefore, the ATP-generating reactions r18 and r25 are multiplied by a Michaelis–Menten term that sharply reduces fluxes below a hypoxic threshold [11] (0.011 mM O_2_):

Term for inhibition by oxygen

(A5) $$O_{2inhibition}=\frac{O_{2}}{O_{2}+0.011}$$

Since oxygen concentration now varies dynamically due to cellular consumption, it is treated as a variable. Its consumption is calculated based on respiratory chain activity, i.e., ATP production. The model of Li and Wang [5] captures the essential mechanisms for the fluxes of interest while simplifying reaction divisions. ATP production differs for NADH and $FADH_{2}$: NADH yields slightly more protons, ultimately producing 2.5 ATP per molecule versus 1.5 ATP for $FADH_{2}$. The model neglects $FADH_{2}$ in the first reaction (r25), accounting for the excess ATP from NADH (1 ATP per “complex II” unit), and in a second step (r27) calculates the remaining ATP (1.5) contributed by both NADH and $FADH_{2}$. This approach reduces the number of variables and equations at the cost of readability. On average, a pyruvate molecule yields 12.5 mitochondrial ATP, with 80% from NADH (10 ATP) and 20% from $FADH_{2}$ (1.5 ATP). Scaling to complex II units gives: $1.5ATP_{FADH_{2}}\cdot 0.2+2.5ATP_{NADH}\cdot 0.8=2.3ATP/\mathrm{complex}\mathrm{II}$. Oxygen consumption from complex II to IV depends on the $NADH/FADH_{2}$ ratio. With NADH, the P/O ratio is 5 (1 ATP per 1/5 O_2_), and with $FADH_{2}$ it is 3 (1 ATP per 1/3 O_2_). Using the same proportions as before: $0.8\times \frac{1}{5}+0.2\times \frac{1}{3}=0.226O_{2}/\mathrm{ATP}$. The final formula for the oxygen consumed can therefore be summarized as follows:

$$\frac{dO_{2}}{dt}=Q_{O_{2}/\mathrm{ATP}}\cdot Q_{\mathrm{ATP}/\mathrm{complex}\mathrm{II}}\cdot {\mathrm{Flux}}_{\mathrm{Complex}\mathrm{II}\to \mathrm{IV}}$$

That is to say:

Oxygen over time

(A6) $$\frac{dO_{2}}{dt}=0.226\times 2.3\times r_{27}$$

*Fluxes entering and leaving the cell*

Lactate transport between the cell and its environment was revised to include proton coupling. In the original model, flux depended solely on the intra/extracellular lactate gradient, whereas monocarboxylate transporters (MCT1 and MCT4) are proton-linked [38]. Thus, transport depends on both lactate and proton gradients, resulting in a dynamic equilibrium between the two.

The transport equations were formulated based on the observations of Xie et al. [6]:

- the intracellular lactate/pyruvate ratio exceeds ∼10 at all pH values;
- pyruvate concentration remains relatively constant above 0.1 mM;
- under acidic extracellular pH, when extracellular lactate exceeds intracellular levels or the lactate/pyruvate ratio decreases, lactate enters the cell;
- at neutral pH, lactate influx remains low even if extracellular lactate is higher.

MCT1 and MCT4 differ thermodynamically, favoring transport in opposite directions. To simplify, a single generic carrier representing overall dynamics was modeled:

Lactate and proton flux

(A7) $$r21=V_{mf_{21}}\cdot \left(r21_{forward}-r21_{reverse}\right)$$

Outward transport of lactate and proton

$$\begin{array}{cc} r21_{forward} & =\frac{1}{e^{2(Lactate_{out}-Lactate)}+1}\cdot \frac{Lactate}{Lactate+0.01}\cdot Regulation_{lac/pyr} \end{array}$$

with $Lactate_{out}$ and Lactate denoting extra- and intracellular lactate concentrations, respectively.

The first term reduces outflow when extracellular lactate approaches or exceeds intracellular levels, and tends to 1 when $Lactate>Lactate_{out}$. The second term prevents efflux when intracellular lactate is nearly zero.

The last regulation term governs the lactate/pyruvate balance:

Regulation of the lactate/pyruvate ratio

(A8) $$Regulation_{lac/pyr}=\frac{1}{1+\frac{1}{{\left(e^{Ratio_{lac/pyr}-10}\right)}^{2}}}\cdot \frac{1}{e^{150(0.1-Pyruvate)}+1}$$

with

(A9) $$Ratio_{lac/pyr}=\frac{Lactate+0.001}{Pyruvate+0.001}$$

Small constants (0.001) prevent singularities when pyruvate →0. The first term drives lactate efflux from 1 (when $Ratio_{lac/pyr}>10$) to 0 below this threshold. The second term increases efflux from 0 to 1 as pyruvate rises above 0.1 mM, maintaining intracellular lactate when pyruvate is scarce. The slope factor (150) adjusts the sharpness of the transition without causing numerical instability.

Incoming transport of lactate and protons

$$\begin{array}{cc} r21_{reverse} & =0.2\cdot (1-\frac{1}{e^{2\cdot (Lactate_{out}-Lactate)}+1})\\ & \cdot \frac{Lactate_{out}}{Lactate_{out}+0.1}\cdot Regulation_{pH_{e}/pH_{i}} \end{array}$$

The first term of the inflow equation is a constant set to 0.2, corresponding to the maximal uptake rate of lactate via MCTs. Given that the maximal outflow rate is normalized to 1, the maximal inflow is only 20% as efficient, according to the experiments of Xie et al. [6]. This parameter could be tuned if necessary, but no dynamic adjustment was required here. The second term mirrors the outflow formulation: it is subtracted from 1, increasing inflow when extracellular lactate approaches or exceeds intracellular levels, and driving it to zero when $Lactate>Lactate_{out}$. The third term suppresses inflow when extracellular lactate approaches zero, preventing uptake in the absence of substrate. The last $Regulation_{pH_{e}/pH_{i}}$ term is defined by:

Regulation of the inward transport of lactate and protons by pH

(A10) $$Regulation_{pH_{e}/pH_{i}}=1-\frac{1}{e^{-2\cdot (pH_{e}-\frac{pH_{i}}{pH_{eq}})}+1}$$

This last term, varying between 0 and 1, promotes the incoming transport of lactate and protons when the difference between $pH_{e}$ and $pH_{i}$ weighted by $pH_{eq}$ is positive. Typically the value of $pH_{eq}$ is 1.1 which allows an equilibrium for an extracellular pH slightly more acidic than the intracellular one. The values of the intracellular pH ($pH_{i}$) are prescribed from experimental data performed on U87 cells exposed to a range of extracellular pH ($pH_{e}$) [39].

Figure A2, shows the result of modeling r21 for $V_{mf_{21}}=1$. The final flow in black is slightly below its maximum speed of 1 when the extracellular lactate is close to 0 because the pyruvate is very close to its minimum concentration and the lactate/pyruvate ratio is not large enough to compensate. Export is therefore important due to a lack of lactate on the outside, but not maximum to avoid depleting all the pyruvate. The pH being sufficiently acidic, the inflow (in red) tends towards its maximum speed of 0.2.

To calculate the concentrations of the different metabolites, all that remains is to add the corresponding incoming and outgoing fluxes (Table A1). For example, for intracellular glucose:

Concentration of intracellular glucose over time

(A11) $$\frac{dGlucose}{dt}=r_{1}-r_{2}$$

For protons, only lactate transport fluxes (r21) significantly affect their dynamics. As mentioned above, each transported lactate molecule is accompanied by a proton, leading to the same lactate and proton variations. However, this equivalence does not hold due to buffering effects. When protons are added, the solution resists pH variation through the equilibrium between weak acids and their conjugate bases. The buffer capacity, which quantifies this resistance, is specific to each solution. Van Slyke’s equation [40] accurately describes the evolution of pH as a function of the existing pH and the number of protons added (via r21). The following expression directly results from its application:

Evolution of the concentration of extracellular H+ ions over time

(A12) $$\frac{dH_{out}^{+}}{dt}=\frac{r_{21}}{1000\cdot (-\frac{-Ka_{lac}\cdot Lactate_{out}}{1000\cdot {\left(H_{out}^{+}+Ka_{lac}\right)}^{2}}+1+\frac{Ka_{water}}{{\left(H_{out}^{+}\right)}^{2}})}$$

with $Ka_{lac}=10^{-3.86}$ mole/L, dissociation constant of lactate and $Ka_{water}=10^{-14}$ mole/L, dissociation constant of water. The constants of 1000 are here to convert molars into millimolars.

*Implementation in PhysiCell*

When multiple cells occupy the same voxel, they read identical concentrations at time *t* and attempt to transport the same fluxes. If nutrient availability is insufficient, concentrations could become locally negative, i.e., cells would absorb more nutrients than available at time *t*. To prevent this, a *voxel debt* system was implemented: instead of instantaneously exchanging matter between cells and voxels, the transfer is distributed until the next metabolic evaluation step. The resulting simulations are equivalent to those obtained with a smaller time step.

*Regulation of gene expression*

The previous section defined the metabolic flux equations, some of which depend directly on specific gene expression levels (e.g., the glucose transporter GLUT1 in reaction r1), or indirectly through upstream regulators (e.g., HIF enhances GLUT1 expression, which then affects r1). The model by Li and Wang [5] includes 13 genes closely interacting with enzymes and metabolites. Their expression levels are normalized to study their dynamics.

Evolution of the expression level of a gene over time

(A13) $$\frac{dX}{dt}=A\cdot H\left(Y\right)-D\cdot X$$

The expression level of each gene *X* increases at a basal rate *A* of 0.005/min (normalized level/min) and is degraded at the same rate *D*. These values are calibrated on measurements carried out in previous studies and specified in the related publications. H(Y) is a Hill function defining the regulation of the gene *Y* on the gene *X*:

(A14) $$H\left(Y\right)=\frac{S^{n}}{S^{n}+Y^{n}}+\gamma \cdot \frac{Y^{n}}{S^{n}+Y^{n}}$$

γ defines the type of regulation (positive > 1, negative < 1 and neutral = 1), *S* the gene level *Y* at which the expression is half (similar to $K_{m}$ of a Michaelis–Menten) and *n* the regulatory force (between 0.025 and 40).

All equations are defined in Supplementary File S1. They remain unchanged from the original publication, except for the HIF equation: instead of a constant degradation rate, we made it oxygen-dependent by introducing the term H(O_2_) in the degradation term.

Gene behavior and regulation are primarily governed by the regulatory strength parameter γ. The closer γ is to 1, the weaker the influence between the elements of a gene–gene, gene–enzyme, or metabolite–gene pair; the further it is from 1, the stronger the influence. In the original model, γ values were randomly generated. To account for metabolic dynamics over time, we introduced inheritance of similar γ values in daughter cells after division.

Initially, all cells share close, literature-calibrated γ values. Upon division, each γ is redrawn from a normal distribution centered on its parent value (maintaining the mode of action, i.e., inhibitory genes cannot exceed 1). This allows gradual epigenomic drift, where small variations dominate but occasional large changes occur. Non-viable configurations lead to cell death, limiting the propagation of unsuitable γ values.
