# Supplementary material for: Beyond the Warburg Effect: Modeling the Dynamic and Context-Dependent Nature of Tumor Metabolism
Source: Cancers (Basel). 2025 Nov 3;17(21):3563. doi: 10.3390/cancers17213563 (PMC12608645; doi:10.3390/cancers17213563)
Supplement: Supplementary file 1 [file cancers-17-03563-s001.zip › cancers-3908708-supplementary.pdf]

File S1. Equations of the model of cell metabolism

The model for cell energy metabolism by Li and Wang (2020) has been modified as described in the *Models* section. The model comprises 56 ODEs, 119 simple parameters and 3 matrices S, γ and n of size 53×53 and 1 vector l_x of size 53. The equations describe the evolution over time of the level of expression of genes, enzymes as well as concentrations of metabolites and values of reaction flows.

Reference

Li W, Wang J. Uncovering the Underlying Mechanisms of Cancer Metabolism though the Landscapes and Probability Flux Quantifications. iScience. 2020 4;23(4):101002. https://doi.org/10.1016/j.isci.2020.101002

$$\frac{d\text{Akt}}{dt} = A_0 \cdot \left(\frac{\text{AMPK}^{n_{1,0}} \cdot \gamma_{1,0}}{\text{AMPK}^{n_{1,0}} + S_{1,0}^{n_{1,0}}} + \frac{S_{1,0}^{n_{1,0}}}{\text{AMPK}^{n_{1,0}} + S_{1,0}^{n_{1,0}}} \right) \cdot \left(\frac{\text{PDK}^{n_{7,0}} \cdot \gamma_{7,0}}{\text{PDK}^{n_{7,0}} + S_{7,0}^{n_{7,0}}} + \frac{S_{7,0}^{n_{7,0}}}{\text{PDK}^{n_{7,0}} + S_{7,0}^{n_{7,0}}} \right)$$
$$\cdot \left(\frac{\text{PI3K}^{n_{8,0}} \cdot \gamma_{8,0}}{\text{PI3K}^{n_{8,0}} + S_{8,0}^{n_{8,0}}} + \frac{S_{8,0}^{n_{8,0}}}{\text{PI3K}^{n_{8,0}} + S_{8,0}^{n_{8,0}}} \right) - D_0 \cdot \text{Akt}$$

$$\frac{d\text{AMPK}}{dt} = A_1 \cdot \left(\frac{\text{RAS}^{n_{10,1}} \cdot \gamma_{10,1}}{\text{RAS}^{n_{10,1}} + S_{10,1}^{n_{10,1}}} + \frac{S_{10,1}^{n_{10,1}}}{\text{RAS}^{n_{10,1}} + S_{10,1}^{n_{10,1}}} \right) \cdot \left(\frac{\text{VEGF}^{n_{12,1}} \cdot \gamma_{12,1}}{\text{VEGF}^{n_{12,1}} + S_{12,1}^{n_{12,1}}} + \frac{S_{12,1}^{n_{12,1}}}{\text{VEGF}^{n_{12,1}} + S_{12,1}^{n_{12,1}}} \right)$$
$$\cdot \left(\frac{\text{HIF}^{n_{3,1}} \cdot \gamma_{3,1}}{\text{HIF}^{n_{3,1}} + S_{3,1}^{n_{3,1}}} + \frac{S_{3,1}^{n_{3,1}}}{\text{HIF}^{n_{3,1}} + S_{3,1}^{n_{3,1}}} \right) \cdot \left(\frac{(\text{R5P}/lx_{42})^{n_{42,1}} \cdot \gamma_{42,1}}{(\text{R5P}/lx_{42})^{n_{42,1}} + S_{42,1}^{n_{42,1}}} + \frac{S_{42,1}^{n_{42,1}}}{(\text{R5P}/lx_{42})^{n_{42,1}} + S_{42,1}^{n_{42,1}}} \right)$$
$$\cdot \left(\frac{\left(\frac{lx_{46} \cdot \text{ATP}}{lx_{48} \cdot \text{AMP}}\right)^{n_{48,1}} \cdot \gamma_{48,1}}{\left(\frac{lx_{46} \cdot \text{ATP}}{lx_{48} \cdot \text{AMP}}\right)^{n_{48,1}} + S_{48,1}^{n_{48,1}}} + \frac{S_{48,1}^{n_{48,1}}}{\left(\frac{lx_{46} \cdot \text{ATP}}{lx_{48} \cdot \text{AMP}}\right)^{n_{48,1}} + S_{48,1}^{n_{48,1}}} \right) - D_1 \cdot \text{AMPK}$$

$$\frac{d\text{cMyc}}{dt} = A_2 \cdot \left(\frac{\text{Akt}^{n_{0,2}} \cdot \gamma_{0,2}}{\text{Akt}^{n_{0,2}} + S_{0,2}^{n_{0,2}}} + \frac{S_{0,2}^{n_{0,2}}}{\text{Akt}^{n_{0,2}} + S_{0,2}^{n_{0,2}}} \right) \cdot \left(\frac{\text{p53}^{n_{6,2}} \cdot \gamma_{6,2}}{\text{p53}^{n_{6,2}} + S_{6,2}^{n_{6,2}}} + \frac{S_{6,2}^{n_{6,2}}}{\text{p53}^{n_{6,2}} + S_{6,2}^{n_{6,2}}} \right)$$
$$\cdot \left(\frac{(\text{ROS}/lx_{52})^{n_{52,2}} \cdot \gamma_{52,2}}{(\text{ROS}/lx_{52})^{n_{52,2}} + S_{52,2}^{n_{52,2}}} + \frac{S_{52,2}^{n_{52,2}}}{(\text{ROS}/lx_{52})^{n_{52,2}} + S_{52,2}^{n_{52,2}}} \right) - D_2 \cdot \text{cMyc}$$

$$\frac{d\text{HIF}}{dt} = A_3 \cdot \left(\frac{\text{RAS}^{n_{10,3}} \cdot \gamma_{10,3}}{\text{RAS}^{n_{10,3}} + S_{10,3}^{n_{10,3}}} + \frac{S_{10,3}^{n_{10,3}}}{\text{RAS}^{n_{10,3}} + S_{10,3}^{n_{10,3}}} \right) \cdot \left(\frac{\text{cMyc}^{n_{2,3}} \cdot \gamma_{2,3}}{\text{cMyc}^{n_{2,3}} + S_{2,3}^{n_{2,3}}} + \frac{S_{2,3}^{n_{2,3}}}{\text{cMyc}^{n_{2,3}} + S_{2,3}^{n_{2,3}}} \right)$$
$$\cdot \left(\frac{\text{mTOR}^{n_{4,3}} \cdot \gamma_{4,3}}{\text{mTOR}^{n_{4,3}} + S_{4,3}^{n_{4,3}}} + \frac{S_{4,3}^{n_{4,3}}}{\text{mTOR}^{n_{4,3}} + S_{4,3}^{n_{4,3}}} \right) \cdot \left(\frac{\text{p53}^{n_{6,3}} \cdot \gamma_{6,3}}{\text{p53}^{n_{6,3}} + S_{6,3}^{n_{6,3}}} + \frac{S_{6,3}^{n_{6,3}}}{\text{p53}^{n_{6,3}} + S_{6,3}^{n_{6,3}}} \right)$$
$$\cdot \left(\frac{\text{PTEN}^{n_{9,3}} \cdot \gamma_{9,3}}{\text{PTEN}^{n_{9,3}} + S_{9,3}^{n_{9,3}}} + \frac{S_{9,3}^{n_{9,3}}}{\text{PTEN}^{n_{9,3}} + S_{9,3}^{n_{9,3}}} \right) \cdot \left(\frac{(\text{Lactate}/lx_{41})^{n_{41,3}} \cdot \gamma_{41,3}}{(\text{Lactate}/lx_{41})^{n_{41,3}} + S_{41,3}^{n_{41,3}}} + \frac{S_{41,3}^{n_{41,3}}}{(\text{Lactate}/lx_{41})^{n_{41,3}} + S_{41,3}^{n_{41,3}}} \right)$$
$$\cdot \left(\frac{(\text{ROS}/lx_{52})^{n_{52,3}} \cdot \gamma_{52,3}}{(\text{ROS}/lx_{52})^{n_{52,3}} + S_{52,3}^{n_{52,3}}} + \frac{S_{52,3}^{n_{52,3}}}{(\text{ROS}/lx_{52})^{n_{52,3}} + S_{52,3}^{n_{52,3}}} \right)$$
$$- D_3 \cdot \text{HIF} \left(\frac{O_2^{n_{O_2,HIF}} \cdot \gamma_{O_2,HIF}}{O_2^{n_{O_2,HIF}} + S_{O_2,HIF}^{n_{O_2,HIF}}} + \frac{S_{O_2,HIF}^{n_{O_2,HIF}}}{O_2^{n_{O_2,HIF}} + S_{O_2,HIF}^{n_{O_2,HIF}}} \right)$$

$$\frac{d\mathrm{mTOR}}{dt} = A_4 \cdot \left(\frac{\mathrm{Akt}^{n_{0,4}} \cdot \gamma_{0,4}}{\mathrm{Akt}^{n_{0,4}} + S_{0,4}^{n_{0,4}}} + \frac{S_{0,4}^{n_{0,4}}}{\mathrm{Akt}^{n_{0,4}} + S_{0,4}^{n_{0,4}}} \right) \cdot \left(\frac{\mathrm{AMPK}^{n_{1,4}} \cdot \gamma_{1,4}}{\mathrm{AMPK}^{n_{1,4}} + S_{1,4}^{n_{1,4}}} + \frac{S_{1,4}^{n_{1,4}}}{\mathrm{AMPK}^{n_{1,4}} + S_{1,4}^{n_{1,4}}} \right)$$
$$\cdot \left(\frac{\mathrm{PI3K}^{n_{8,4}} \cdot \gamma_{8,4}}{\mathrm{PI3K}^{n_{8,4}} + S_{8,4}^{n_{8,4}}} + \frac{S_{8,4}^{n_{8,4}}}{\mathrm{PI3K}^{n_{8,4}} + S_{8,4}^{n_{8,4}}} \right) - D_4 \cdot \mathrm{mTOR}$$

$$\frac{d\mathrm{NOX}}{dt} = A_5 \cdot \left(\frac{\mathrm{RAS}^{n_{10,5}} \cdot \gamma_{10,5}}{\mathrm{RAS}^{n_{10,5}} + S_{10,5}^{n_{10,5}}} + \frac{S_{10,5}^{n_{10,5}}}{\mathrm{RAS}^{n_{10,5}} + S_{10,5}^{n_{10,5}}} \right) \cdot \left(\frac{\mathrm{AMPK}^{n_{1,5}} \cdot \gamma_{1,5}}{\mathrm{AMPK}^{n_{1,5}} + S_{1,5}^{n_{1,5}}} + \frac{S_{1,5}^{n_{1,5}}}{\mathrm{AMPK}^{n_{1,5}} + S_{1,5}^{n_{1,5}}} \right)$$
$$\cdot \left(\frac{\mathrm{HIF}^{n_{3,5}} \cdot \gamma_{3,5}}{\mathrm{HIF}^{n_{3,5}} + S_{3,5}^{n_{3,5}}} + \frac{S_{3,5}^{n_{3,5}}}{\mathrm{HIF}^{n_{3,5}} + S_{3,5}^{n_{3,5}}} \right) \cdot \left(\frac{(\mathrm{ROS}/lx_{52})^{n_{52,5}} \cdot \gamma_{52,5}}{(\mathrm{ROS}/lx_{52})^{n_{52,5}} + S_{52,5}^{n_{52,5}}} + \frac{S_{52,5}^{n_{52,5}}}{(\mathrm{ROS}/lx_{52})^{n_{52,5}} + S_{52,5}^{n_{52,5}}} \right) - D_5 \cdot \mathrm{NOX}$$

$$\frac{d\mathrm{p53}}{dt} = A_6 \cdot \left(\frac{\mathrm{Akt}^{n_{0,6}} \cdot \gamma_{0,6}}{\mathrm{Akt}^{n_{0,6}} + S_{0,6}^{n_{0,6}}} + \frac{S_{0,6}^{n_{0,6}}}{\mathrm{Akt}^{n_{0,6}} + S_{0,6}^{n_{0,6}}} \right) \cdot \left(\frac{\mathrm{SOD}^{n_{11,6}} \cdot \gamma_{11,6}}{\mathrm{SOD}^{n_{11,6}} + S_{11,6}^{n_{11,6}}} + \frac{S_{11,6}^{n_{11,6}}}{\mathrm{SOD}^{n_{11,6}} + S_{11,6}^{n_{11,6}}} \right)$$
$$\cdot \left(\frac{\mathrm{PTEN}^{n_{9,6}} \cdot \gamma_{9,6}}{\mathrm{PTEN}^{n_{9,6}} + S_{9,6}^{n_{9,6}}} + \frac{S_{9,6}^{n_{9,6}}}{\mathrm{PTEN}^{n_{9,6}} + S_{9,6}^{n_{9,6}}} \right) - D_6 \cdot \mathrm{p53}$$

$$\frac{d\mathrm{PDK}}{dt} = A_7 \cdot \left(\frac{\mathrm{HIF}^{n_{3,7}} \cdot \gamma_{3,7}}{\mathrm{HIF}^{n_{3,7}} + S_{3,7}^{n_{3,7}}} + \frac{S_{3,7}^{n_{3,7}}}{\mathrm{HIF}^{n_{3,7}} + S_{3,7}^{n_{3,7}}} \right) \cdot \left(\frac{\mathrm{p53}^{n_{6,7}} \cdot \gamma_{6,7}}{\mathrm{p53}^{n_{6,7}} + S_{6,7}^{n_{6,7}}} + \frac{S_{6,7}^{n_{6,7}}}{\mathrm{p53}^{n_{6,7}} + S_{6,7}^{n_{6,7}}} \right)$$
$$\cdot \left(\frac{\left(\frac{lx_{47} \cdot \mathrm{ATP}}{lx_{48} \cdot \mathrm{ADP}}\right)^{n_{48,7}} \cdot \gamma_{48,7}}{\left(\frac{lx_{47} \cdot \mathrm{ATP}}{lx_{48} \cdot \mathrm{ADP}}\right)^{n_{48,7}} + S_{48,7}^{n_{48,7}}} + \frac{S_{48,7}^{n_{48,7}}}{\left(\frac{lx_{47} \cdot \mathrm{ATP}}{lx_{48} \cdot \mathrm{ADP}}\right)^{n_{48,7}} + S_{48,7}^{n_{48,7}}} \right) - D_7 \cdot \mathrm{PDK}$$

$$\frac{d\mathrm{PI3K}}{dt} = A_8 \cdot \left(\frac{\mathrm{RAS}^{n_{10,8}} \cdot \gamma_{10,8}}{\mathrm{RAS}^{n_{10,8}} + S_{10,8}^{n_{10,8}}} + \frac{S_{10,8}^{n_{10,8}}}{\mathrm{RAS}^{n_{10,8}} + S_{10,8}^{n_{10,8}}} \right) \cdot \left(\frac{\mathrm{PTEN}^{n_{9,8}} \cdot \gamma_{9,8}}{\mathrm{PTEN}^{n_{9,8}} + S_{9,8}^{n_{9,8}}} + \frac{S_{9,8}^{n_{9,8}}}{\mathrm{PTEN}^{n_{9,8}} + S_{9,8}^{n_{9,8}}} \right)$$
$$\cdot \left(\frac{(\mathrm{Lactate}/lx_{41})^{n_{41,8}} \cdot \gamma_{41,8}}{(\mathrm{Lactate}/lx_{41})^{n_{41,8}} + S_{41,8}^{n_{41,8}}} + \frac{S_{41,8}^{n_{41,8}}}{(\mathrm{Lactate}/lx_{41})^{n_{41,8}} + S_{41,8}^{n_{41,8}}} \right)$$
$$\cdot \left(\frac{(\mathrm{ROS}/lx_{52})^{n_{52,8}} \cdot \gamma_{52,8}}{(\mathrm{ROS}/lx_{52})^{n_{52,8}} + S_{52,8}^{n_{52,8}}} + \frac{S_{52,8}^{n_{52,8}}}{(\mathrm{ROS}/lx_{52})^{n_{52,8}} + S_{52,8}^{n_{52,8}}} \right) - D_8 \cdot \mathrm{PI3K}$$

$$\frac{d\text{PTEN}}{dt} = A_9 \cdot \left(\frac{\text{p53}^{n_{6,9}} \cdot \gamma_{6,9}}{\text{p53}^{n_{6,9}} + S_{6,9}^{n_{6,9}}} + \frac{S_{6,9}^{n_{6,9}}}{\text{p53}^{n_{6,9}} + S_{6,9}^{n_{6,9}}} \right) - D_9 \cdot \text{PTEN}$$

$$\frac{d\text{RAS}}{dt} = A_{10} \cdot \left(\frac{\text{VEGF}^{n_{12,10}} \cdot \gamma_{12,10}}{\text{VEGF}^{n_{12,10}} + S_{12,10}^{n_{12,10}}} + \frac{S_{12,10}^{n_{12,10}}}{\text{VEGF}^{n_{12,10}} + S_{12,10}^{n_{12,10}}} \right)$$
$$\cdot \left(\frac{\left(\text{ROS}/lx_{52}\right)^{n_{52,10}} \cdot \gamma_{52,10}}{\left(\text{ROS}/lx_{52}\right)^{n_{52,10}} + S_{52,10}^{n_{52,10}}} + \frac{S_{52,10}^{n_{52,10}}}{\left(\text{ROS}/lx_{52}\right)^{n_{52,10}} + S_{52,10}^{n_{52,10}}} \right) - D_{10} \cdot \text{RAS}$$

$$\frac{d\text{SOD}}{dt} = A_{11} \cdot \left(\frac{\text{VEGF}^{n_{12,11}} \cdot \gamma_{12,11}}{\text{VEGF}^{n_{12,11}} + S_{12,11}^{n_{12,11}}} + \frac{S_{12,11}^{n_{12,11}}}{\text{VEGF}^{n_{12,11}} + S_{12,11}^{n_{12,11}}} \right)$$
$$\cdot \left(\frac{\left(\text{ROS}/lx_{52}\right)^{n_{52,11}} \cdot \gamma_{52,11}}{\left(\text{ROS}/lx_{52}\right)^{n_{52,11}} + S_{52,11}^{n_{52,11}}} + \frac{S_{52,11}^{n_{52,11}}}{\left(\text{ROS}/lx_{52}\right)^{n_{52,11}} + S_{52,11}^{n_{52,11}}} \right) - D_{11} \cdot \text{SOD}$$

$$\frac{d\text{VEGF}}{dt} = A_{12} \cdot \left(\frac{\text{cMyc}^{n_{2,12}} \cdot \gamma_{2,12}}{\text{cMyc}^{n_{2,12}} + S_{2,12}^{n_{2,12}}} + \frac{S_{2,12}^{n_{2,12}}}{\text{cMyc}^{n_{2,12}} + S_{2,12}^{n_{2,12}}} \right)$$
$$\cdot \left(\frac{\text{HIF}^{n_{3,12}} \cdot \gamma_{3,12}}{\text{HIF}^{n_{3,12}} + S_{3,12}^{n_{3,12}}} + \frac{S_{3,12}^{n_{3,12}}}{\text{HIF}^{n_{3,12}} + S_{3,12}^{n_{3,12}}} \right) \cdot \left(\frac{\text{PI3K}^{n_{8,12}} \cdot \gamma_{8,12}}{\text{PI3K}^{n_{8,12}} + S_{8,12}^{n_{8,12}}} + \frac{S_{8,12}^{n_{8,12}}}{\text{PI3K}^{n_{8,12}} + S_{8,12}^{n_{8,12}}} \right)$$
$$\cdot \left(\frac{\left(\text{Lactate}/lx_{41}\right)^{n_{41,12}} \cdot \gamma_{41,12}}{\left(\text{Lactate}/lx_{41}\right)^{n_{41,12}} + S_{41,12}^{n_{41,12}}} + \frac{S_{41,12}^{n_{41,12}}}{\left(\text{Lactate}/lx_{41}\right)^{n_{41,12}} + S_{41,12}^{n_{41,12}}} \right)$$
$$\cdot \left(\frac{\left(\text{ROS}/lx_{52}\right)^{n_{52,12}} \cdot \gamma_{52,12}}{\left(\text{ROS}/lx_{52}\right)^{n_{52,12}} + S_{52,12}^{n_{52,12}}} + \frac{S_{52,12}^{n_{52,12}}}{\left(\text{ROS}/lx_{52}\right)^{n_{52,12}} + S_{52,12}^{n_{52,12}}} \right) - D_{12} \cdot \text{VEGF}$$

$$\frac{d\text{GluT1}}{dt} = A_{13} \cdot \left(\frac{\text{Akt}^{n_{0,13}} \cdot \gamma_{0,13}}{\text{Akt}^{n_{0,13}} + S_{0,13}^{n_{0,13}}} + \frac{S_{0,13}^{n_{0,13}}}{\text{Akt}^{n_{0,13}} + S_{0,13}^{n_{0,13}}} \right) \cdot \left(\frac{\text{cMyc}^{n_{2,13}} \cdot \gamma_{2,13}}{\text{cMyc}^{n_{2,13}} + S_{2,13}^{n_{2,13}}} + \frac{S_{2,13}^{n_{2,13}}}{\text{cMyc}^{n_{2,13}} + S_{2,13}^{n_{2,13}}} \right)$$
$$\cdot \left(\frac{\text{HIF}^{n_{3,13}} \cdot \gamma_{3,13}}{\text{HIF}^{n_{3,13}} + S_{3,13}^{n_{3,13}}} + \frac{S_{3,13}^{n_{3,13}}}{\text{HIF}^{n_{3,13}} + S_{3,13}^{n_{3,13}}} \right) \cdot \left(\frac{\text{p53}^{n_{6,13}} \cdot \gamma_{6,13}}{\text{p53}^{n_{6,13}} + S_{6,13}^{n_{6,13}}} + \frac{S_{6,13}^{n_{6,13}}}{\text{p53}^{n_{6,13}} + S_{6,13}^{n_{6,13}}} \right) - D_{13} \cdot \text{GluT1}$$

$$\frac{d\text{HK}}{dt} = A_{14} \cdot \left(\frac{\text{Akt}^{n_{0,14}} \cdot \gamma_{0,14}}{\text{Akt}^{n_{0,14}} + S_{0,14}^{n_{0,14}}} + \frac{S_{0,14}^{n_{0,14}}}{\text{Akt}^{n_{0,14}} + S_{0,14}^{n_{0,14}}} \right) \cdot \left(\frac{\text{HIF}^{n_{3,14}} \cdot \gamma_{3,14}}{\text{HIF}^{n_{3,14}} + S_{3,14}^{n_{3,14}}} + \frac{S_{3,14}^{n_{3,14}}}{\text{HIF}^{n_{3,14}} + S_{3,14}^{n_{3,14}}} \right) - D_{14} \cdot \text{HK}$$

$$\frac{d\text{G6PD}}{dt} = A_{15} \cdot \left(\frac{\text{HIF}^{n_{3,15}} \cdot \gamma_{3,15}}{\text{HIF}^{n_{3,15}} + S_{3,15}^{n_{3,15}}} + \frac{S_{3,15}^{n_{3,15}}}{\text{HIF}^{n_{3,15}} + S_{3,15}^{n_{3,15}}} \right) \cdot \left(\frac{\text{p53}^{n_{6,15}} \cdot \gamma_{6,15}}{\text{p53}^{n_{6,15}} + S_{6,15}^{n_{6,15}}} + \frac{S_{6,15}^{n_{6,15}}}{\text{p53}^{n_{6,15}} + S_{6,15}^{n_{6,15}}} \right) - D_{15} \cdot \text{G6PD}$$

$$\frac{d\text{GPI}}{dt} = A_{16} \cdot \left(\frac{\text{HIF}^{n_{3,16}} \cdot \gamma_{3,16}}{\text{HIF}^{n_{3,16}} + S_{3,16}^{n_{3,16}}} + \frac{S_{3,16}^{n_{3,16}}}{\text{HIF}^{n_{3,16}} + S_{3,16}^{n_{3,16}}} \right) - D_{16} \cdot \text{GPI}$$

$$\begin{aligned} \frac{d\text{PFKFB2}}{dt} = A_{17} \cdot & \left(\frac{\text{Akt}^{n_{0,17}} \cdot \gamma_{0,17}}{\text{Akt}^{n_{0,17}} + S_{0,17}^{n_{0,17}}} + \frac{S_{0,17}^{n_{0,17}}}{\text{Akt}^{n_{0,17}} + S_{0,17}^{n_{0,17}}} \right) \cdot \left(\frac{\text{AMPK}^{n_{1,17}} \cdot \gamma_{1,17}}{\text{AMPK}^{n_{1,17}} + S_{1,17}^{n_{1,17}}} + \frac{S_{1,17}^{n_{1,17}}}{\text{AMPK}^{n_{1,17}} + S_{1,17}^{n_{1,17}}} \right) \\ & \cdot \left(\frac{\text{HIF}^{n_{3,17}} \cdot \gamma_{3,17}}{\text{HIF}^{n_{3,17}} + S_{3,17}^{n_{3,17}}} + \frac{S_{3,17}^{n_{3,17}}}{\text{HIF}^{n_{3,17}} + S_{3,17}^{n_{3,17}}} \right) - D_{17} \cdot \text{PFKFB2} \end{aligned}$$

$$\frac{d\text{PFK1}}{dt} = A_{18} \cdot \left(\frac{\text{RAS}^{n_{10,18}} \cdot \gamma_{10,18}}{\text{RAS}^{n_{10,18}} + S_{10,18}^{n_{10,18}}} + \frac{S_{10,18}^{n_{10,18}}}{\text{RAS}^{n_{10,18}} + S_{10,18}^{n_{10,18}}} \right) \cdot \left(\frac{\text{HIF}^{n_{3,18}} \cdot \gamma_{3,18}}{\text{HIF}^{n_{3,18}} + S_{3,18}^{n_{3,18}}} + \frac{S_{3,18}^{n_{3,18}}}{\text{HIF}^{n_{3,18}} + S_{3,18}^{n_{3,18}}} \right) - D_{18} \cdot \text{PFK1}$$

$$\frac{d\text{ALD}}{dt} = A_{19} \cdot \left(\frac{\text{HIF}^{n_{3,19}} \cdot \gamma_{3,19}}{\text{HIF}^{n_{3,19}} + S_{3,19}^{n_{3,19}}} + \frac{S_{3,19}^{n_{3,19}}}{\text{HIF}^{n_{3,19}} + S_{3,19}^{n_{3,19}}} \right) - D_{19} \cdot \text{ALD}$$

$$\frac{d\text{TPI}}{dt} = A_{20} \cdot \left(\frac{\text{HIF}^{n_{3,20}} \cdot \gamma_{3,20}}{\text{HIF}^{n_{3,20}} + S_{3,20}^{n_{3,20}}} + \frac{S_{3,20}^{n_{3,20}}}{\text{HIF}^{n_{3,20}} + S_{3,20}^{n_{3,20}}} \right) - D_{20} \cdot \text{TPI}$$

$$\frac{d\text{GAPDH}}{dt} = A_{21} \cdot \left(\frac{\text{HIF}^{n_{3,21}} \cdot \gamma_{3,21}}{\text{HIF}^{n_{3,21}} + S_{3,21}^{n_{3,21}}} + \frac{S_{3,21}^{n_{3,21}}}{\text{HIF}^{n_{3,21}} + S_{3,21}^{n_{3,21}}} \right) - D_{21} \cdot \text{GAPDH}$$

$$\frac{d\mathrm{PGK}}{dt} = A_{22} \cdot \left(\frac{\mathrm{HIF}^{n_{3,22}} \cdot \gamma_{3,22}}{\mathrm{HIF}^{n_{3,22}} + S_{3,22}^{n_{3,22}}} + \frac{S_{3,22}^{n_{3,22}}}{\mathrm{HIF}^{n_{3,22}} + S_{3,22}^{n_{3,22}}} \right) - D_{22} \cdot \mathrm{PGK}$$

$$\frac{d\mathrm{PHGDH}}{dt} = A_{23} - D_{23} \cdot \mathrm{PHGDH}$$

$$\frac{d\mathrm{PGAM}}{dt} = A_{24} \cdot \left(\frac{\mathrm{HIF}^{n_{3,24}} \cdot \gamma_{3,24}}{\mathrm{HIF}^{n_{3,24}} + S_{3,24}^{n_{3,24}}} + \frac{S_{3,24}^{n_{3,24}}}{\mathrm{HIF}^{n_{3,24}} + S_{3,24}^{n_{3,24}}} \right) - D_{24} \cdot \mathrm{PGAM}$$

$$\frac{d\mathrm{ENO}}{dt} = A_{25} \cdot \left(\frac{\mathrm{HIF}^{n_{3,25}} \cdot \gamma_{3,25}}{\mathrm{HIF}^{n_{3,25}} + S_{3,25}^{n_{3,25}}} + \frac{S_{3,25}^{n_{3,25}}}{\mathrm{HIF}^{n_{3,25}} + S_{3,25}^{n_{3,25}}} \right) - D_{25} \cdot \mathrm{ENO}$$

$$\frac{d\mathrm{PKM2}}{dt} = A_{26} \cdot \left(\frac{\mathrm{HIF}^{n_{3,26}} \cdot \gamma_{3,26}}{\mathrm{HIF}^{n_{3,26}} + S_{3,26}^{n_{3,26}}} + \frac{S_{3,26}^{n_{3,26}}}{\mathrm{HIF}^{n_{3,26}} + S_{3,26}^{n_{3,26}}} \right) - D_{26} \cdot \mathrm{PKM2}$$

$$\frac{d\mathrm{PDH}}{dt} = A_{27} \cdot \left(\frac{\mathrm{PDK}^{n_{7,27}} \cdot \gamma_{7,27}}{\mathrm{PDK}^{n_{7,27}} + S_{7,27}^{n_{7,27}}} + \frac{S_{7,27}^{n_{7,27}}}{\mathrm{PDK}^{n_{7,27}} + S_{7,27}^{n_{7,27}}} \right) \cdot \left(\frac{\mathrm{PTEN}^{n_{9,27}} \cdot \gamma_{9,27}}{\mathrm{PTEN}^{n_{9,27}} + S_{9,27}^{n_{9,27}}} + \frac{S_{9,27}^{n_{9,27}}}{\mathrm{PTEN}^{n_{9,27}} + S_{9,27}^{n_{9,27}}} \right) - D_{27} \cdot \mathrm{PDH}$$

$$\begin{aligned}\frac{d\mathrm{ACC}}{dt} = A_{28} &\cdot \left(\frac{\mathrm{AMPK}^{n_{1,28}} \cdot \gamma_{1,28}}{\mathrm{AMPK}^{n_{1,28}} + S_{1,28}^{n_{1,28}}} + \frac{S_{1,28}^{n_{1,28}}}{\mathrm{AMPK}^{n_{1,28}} + S_{1,28}^{n_{1,28}}} \right) \\ &\cdot \left(\frac{\left(\mathrm{G6P}/lx_{31}\right)^{n_{31,28}} \cdot \gamma_{31,28}}{\left(\mathrm{G6P}/lx_{31}\right)^{n_{31,28}} + S_{31,28}^{n_{31,28}}} + \frac{S_{31,28}^{n_{31,28}}}{\left(\mathrm{G6P}/lx_{31}\right)^{n_{31,28}} + S_{31,28}^{n_{31,28}}} \right) \\ &\cdot \left(\frac{\left(\mathrm{F26BP}/lx_{43}\right)^{n_{43,28}} \cdot \gamma_{43,28}}{\left(\mathrm{F26BP}/lx_{43}\right)^{n_{43,28}} + S_{43,28}^{n_{43,28}}} + \frac{S_{43,28}^{n_{43,28}}}{\left(\mathrm{F26BP}/lx_{43}\right)^{n_{43,28}} + S_{43,28}^{n_{43,28}}} \right) - D_{28} \cdot \mathrm{ACC}\end{aligned}$$

$$\frac{d\mathrm{LDH}}{dt} = A_{29} \cdot \left(\frac{\mathrm{cMyc}^{n_{2,29}} \cdot \gamma_{2,29}}{\mathrm{cMyc}^{n_{2,29}} + S_{2,29}^{n_{2,29}}} + \frac{S_{2,29}^{n_{2,29}}}{\mathrm{cMyc}^{n_{2,29}} + S_{2,29}^{n_{2,29}}} \right) \cdot \left(\frac{\mathrm{HIF}^{n_{3,29}} \cdot \gamma_{3,29}}{\mathrm{HIF}^{n_{3,29}} + S_{3,29}^{n_{3,29}}} + \frac{S_{3,29}^{n_{3,29}}}{\mathrm{HIF}^{n_{3,29}} + S_{3,29}^{n_{3,29}}} \right) - D_{29} \cdot \mathrm{LDH}$$

$$\frac{d\text{Glucose}}{dt} = r_1 - r_2$$

$$\frac{d\text{G6P}}{dt} = -r_{13} + r_2 - r_3$$

$$\frac{d\text{F6P}}{dt} = -r_{16} + 2 \cdot r_{22} + r_3 - r_4$$

$$\frac{d\text{FBP}}{dt} = r_4 - r_5$$

$$\frac{d\text{G3P}}{dt} = r_{22} + r_5 + r_6 - r_7$$

$$\frac{d\text{DHAP}}{dt} = r_5 - r_6$$

$$\frac{d\text{13BPG}}{dt} = r_7 - r_8$$

$$\frac{d\text{3PG}}{dt} = -r_{17} + r_8 - r_9$$

$$\frac{d\text{2PG}}{dt} = -r_{10} + r_9$$

$$\frac{d\text{PEP}}{dt} = r_{10} - r_{11}$$

$$\frac{d\text{Pyruvate}}{dt} = r_{11} - r_{12} - r_{18}$$

$$\frac{d\text{Lactate}}{dt} = r_{12} - r_{21}$$

$$\frac{d\text{R5P}}{dt} = r_{13} - 3 \cdot r_{22} - r_{23}$$

$$\frac{d\text{F26BP}}{dt} = r_{16}$$

$$\frac{d\text{Serine}}{dt} = r_{17} - r_{24}$$

$$\frac{d\text{Citrate}}{dt} = r_{18} - r_{19} - r_{26} - r_{30}$$

$$\frac{d\text{AMP}}{dt} = -r_{15}$$

$$\frac{d\text{ADP}}{dt} = -r_{11} + r_{14} + 2 \cdot r_{15} - r_{18} + 3 \cdot r_{19} + r_2 - r_{25} - 3 \cdot r_{26} - 1{,}5 \cdot r_{27} + r_4 - r_8$$

$$\frac{d\text{ATP}}{dt} = r_{11} - r_{14} - r_{15} + r_{18} - 3 \cdot r_{19} - r_2 + r_{25} + 3 \cdot r_{26} + 1{,}5 \cdot r_{27} - r_4 + r_8$$

$$\frac{d\text{NAD}}{dt} = r_{12} + r_{25} - r_7$$

$$\frac{d\text{NADH}}{dt} = -r_{12} - r_{25} + r_7$$

$$\frac{d\text{complex2}}{dt} = r_{18} - r_{19} + r_{25} + 4 \cdot r_{26} - r_{27} - r_{28}$$

$$\frac{d\text{ROS}}{dt} = -r_{20} + r_{28} + r_{29}$$

$$\frac{d\mathrm{H}^{+}_{\mathrm{out}}}{dt} = \frac{r_{21}}{1000 \cdot \left(-\frac{-Ka_{lac} \cdot \mathrm{Lactate}_{\mathrm{out}}}{1000 \cdot (H_{out} + Ka_{lac})^{2}} + 1 + \frac{Ka_{water}}{H_{out}^{2}} \right)}$$

$$\frac{dO_2}{dt} = 0.226 \cdot 2.3 \cdot r_{27}$$

$$r1 = \frac{V_{mf_1} \cdot \mathrm{GluT1} \cdot \left(Glu_{out} - \frac{\mathrm{Glucose}}{K_{eq_1}} \right)}{Glu_{out} + K_{gluout_1} \cdot \left(1 + \frac{\mathrm{Glucose}}{K_{gluin_1}} \right)}$$

$$r2 = \frac{V_{m_2} \cdot pH_{inhibition} \cdot \mathrm{HK} \cdot \left(\mathrm{Glucose} \cdot \mathrm{ATP} - \frac{\mathrm{G6P}}{K_{eq_2}} \cdot \mathrm{ADP} \right)}{K_{a_2} \cdot K_{b_2} \cdot \left(1 + \frac{\mathrm{ADP}}{K_{q_2}} + \frac{\mathrm{G6P}}{K_{p_2}} + \frac{\mathrm{G6P} \cdot \mathrm{ADP}}{K_{p_2} \cdot K_{q_2}} + \frac{\mathrm{ATP}}{K_{b_2}} + \frac{\mathrm{G6P} \cdot \mathrm{ATP}}{K_{b_2} \cdot K_{p_2}} + \frac{\mathrm{Glucose}}{K_{a_2}} + \frac{\mathrm{Glucose} \cdot \mathrm{ADP}}{K_{a_2} \cdot K_{q_2}} + \frac{\mathrm{Glucose} \cdot \mathrm{ATP}}{K_{a_2} \cdot K_{b_2}} \right)}$$

$$r3 = \frac{pH_{inhibition} \cdot \left(\frac{V_{mf_3}}{K_{g6p_3}} \cdot \mathrm{GPI} \cdot \mathrm{G6P} - \frac{V_{mr_3}}{K_{f6p_3}} \cdot \mathrm{GPI} \cdot \mathrm{F6P} \right)}{\frac{Ery4P}{K_{ery4p_3}} + 1 + \frac{bx6PG}{K_{pg_3}} + \frac{\mathrm{G6P}}{K_{g6p_3}} + \frac{\mathrm{FBP}}{K_{fbp_3}} + \frac{\mathrm{F6P}}{K_{f6p_3}}}$$

$$r4 = \frac{V_{m_4} \cdot pH_{inhibition} \cdot \mathrm{PFK1} \cdot \mathrm{F6P}}{K_{atp_4} \cdot \left(1 + \frac{\mathrm{F6P}}{K_{atp_4}}\right) \cdot \left(1 + \frac{\mathrm{F26BP}}{K_{f26bp_4} \cdot \alpha_4}\right)} \cdot \left(1 + \frac{\beta_4 \cdot \mathrm{F26BP}}{K_{f26bp_4} \cdot \alpha_4}\right) \cdot \left(\frac{\mathrm{ATP} \cdot \left(1 + \frac{\mathrm{F26BP}}{K_{f26bp_4} \cdot \alpha_4}\right) \cdot \left(1 + \frac{\mathrm{ATP} \cdot \left(1 + \frac{\mathrm{F26BP}}{K_{f26bp_4} \cdot \alpha_4}\right)}{K_{f6p_4} \cdot \left(1 + \frac{\mathrm{F26BP}}{K_{f26bp_4}}\right)}\right)^3}{K_{f6p_4} \cdot \left(1 + \frac{\mathrm{F26BP}}{K_{f26bp_4}}\right) \cdot \left(\frac{L_4 \cdot \left(\frac{Citrate}{K_{cit_4}} + 1\right)^4}{\left(1 + \frac{\mathrm{F26BP}}{K_{f26bp_4}}\right)^4} \cdot \left(1 + \frac{\mathrm{F6P}}{Ki_{atp_4}}\right)^4 + \left(1 + \frac{\mathrm{ATP} \cdot \left(1 + \frac{\mathrm{F26BP}}{K_{f26bp_4} \cdot \alpha_4}\right)}{K_{f6p_4} \cdot \left(1 + \frac{\mathrm{F26BP}}{K_{f26bp_4}}\right)}\right)^4 \right)} - \frac{\mathrm{FBP} \cdot \mathrm{ADP}}{K_{adp_4} \cdot K_{eq_4} \cdot K_{fbp_4} \cdot \left(1 + \frac{\mathrm{ADP}}{K_{fbp_4}} + \frac{\mathrm{FBP}}{K_{adp_4}} + \frac{\mathrm{FBP} \cdot \mathrm{ADP}}{K_{adp_4} \cdot K_{fbp_4}}\right)} \right)$$

$$r5 = \frac{pH_{inhibition} \cdot \left(\frac{V_{mf_5}}{K_{fbp_5}} \cdot \mathrm{ALD} \cdot \mathrm{FBP} - \frac{V_{mr_5} \cdot \mathrm{ALD} \cdot \mathrm{G3P}}{K_{dhap_5} \cdot K_{g3p_5}} \cdot \mathrm{DHAP}\right)}{1 + \frac{\mathrm{G3P}}{K_{g3p_5}} + \frac{\mathrm{FBP}}{K_{fbp_5}} + \frac{\mathrm{DHAP}}{K_{dhap_5}} + \frac{\mathrm{G3P} \cdot \mathrm{DHAP}}{K_{dhap_5} \cdot K_{g3p_5}}}$$

$$r6 = pH_{inhibition} \cdot \left(V_{f_6} \cdot \mathrm{TPI} \cdot \mathrm{DHAP} - \frac{V_{r_6} \cdot \mathrm{TPI} \cdot \mathrm{G3P}}{K_{mp_6} \cdot K_{ms_6} + K_{mp_6} \cdot \mathrm{DHAP} + K_{ms_6} \cdot \mathrm{G3P}}\right)$$

$$r7 = \frac{pH_{inhibition} \cdot \left(\frac{Pi \cdot V_{mf_7} \cdot \mathrm{GAPDH} \cdot \mathrm{G3P} \cdot \mathrm{NAD}}{K_{g3p_7} \cdot K_{nad_7} \cdot K_{p_7}} - \frac{V_{mr_7} \cdot \mathrm{GAPDH} \cdot \mathrm{13BPG}}{K_{dpg_7} \cdot K_{nadh_7}} \cdot \mathrm{NADH}\right)}{1 + \frac{\mathrm{NADH}}{K_{nadh_7}} + \frac{\mathrm{NAD}}{K_{nad_7}} + \frac{\mathrm{G3P} \cdot \mathrm{NAD}}{K_{g3p_7} \cdot K_{nad_7}} + \frac{Pi \cdot \mathrm{G3P} \cdot \mathrm{NAD}}{K_{g3p_7} \cdot K_{nad_7} \cdot K_{p_7}} + \frac{\mathrm{13BPG} \cdot \mathrm{NADH}}{K_{dpg_7} \cdot K_{nadh_7}}}$$

$$r8 = \frac{-\frac{V_{mr_8} \cdot \mathrm{PGK} \cdot \mathrm{3PG} \cdot \mathrm{ATP}}{K_{p_8} \cdot K_{q_8} \cdot \beta_8} + \frac{V_{mf_8} \cdot \mathrm{PGK} \cdot \mathrm{13BPG} \cdot \mathrm{ADP}}{K_{a_8} \cdot K_{b_8} \cdot \alpha_8}}{1 + \frac{\mathrm{ATP}}{K_{q_8}} + \frac{\mathrm{3PG}}{K_{p_8}} + \frac{\mathrm{3PG} \cdot \mathrm{ATP}}{K_{p_8} \cdot K_{q_8} \cdot \beta_8} + \frac{\mathrm{ADP}}{K_{b_8}} + \frac{\mathrm{13BPG}}{K_{a_8}} + \frac{\mathrm{13BPG} \cdot \mathrm{ADP}}{K_{a_8} \cdot K_{b_8} \cdot \alpha_8}}$$

$$r9 = V_{f_9} \cdot \mathrm{PGAM} \cdot \mathrm{3PG} - \frac{V_{r_9} \cdot \mathrm{PGAM} \cdot \mathrm{2PG}}{K_{mp_9} \cdot K_{ms_9} + K_{mp_9} \cdot \mathrm{3PG} + K_{ms_9} \cdot \mathrm{2PG}}$$

$$r10 = pH_{inhibition} \cdot \left(V_{f_{10}} \cdot \mathrm{ENO} \cdot \mathrm{2PG} - \frac{V_{r_{10}} \cdot \mathrm{ENO} \cdot \mathrm{PEP}}{K_{mp_{10}} \cdot K_{ms_{10}} + K_{mp_{10}} \cdot \mathrm{2PG} + K_{ms_{10}} \cdot \mathrm{PEP}} \right)$$

$$r11 = \frac{V_{max_{11}} \cdot pH_{inhibition} \cdot \mathrm{PKM2} \cdot \left(\frac{\mathrm{PEP} \cdot \mathrm{ADP}}{K_{adp_{11}} \cdot K_{pep_{11}}} - \frac{\mathrm{Pyruvate} \cdot \mathrm{ATP}}{K_{adp_{11}} \cdot K_{eq_{11}} \cdot K_{pep_{11}}} \right)}{\left(1 + \frac{\mathrm{ATP}}{K_{atp_{11}}} + \frac{\mathrm{ADP}}{K_{adp_{11}}}\right) \cdot \left(1 + \frac{\mathrm{Pyruvate}}{K_{pyr_{11}}} + \frac{\mathrm{PEP}}{K_{pep_{11}}}\right)}$$

$$r12 = \frac{-\frac{V_{mr_{12}} \cdot \mathrm{LDH} \cdot \mathrm{Lactate} \cdot \mathrm{NAD}}{K_{p_{12}} \cdot K_{q_{12}} \cdot \beta_{12}} + \frac{V_{mf_{12}} \cdot \mathrm{LDH} \cdot \mathrm{Pyruvate} \cdot \mathrm{NADH}}{K_{a_{12}} \cdot K_{b_{12}} \cdot \alpha_{12}}}{1 + \frac{\mathrm{NAD}}{K_{q_{12}}} + \frac{\mathrm{Lactate}}{K_{p_{12}}} + \frac{\mathrm{Lactate} \cdot \mathrm{NAD}}{K_{p_{12}} \cdot K_{q_{12}} \cdot \beta_{12}} + \frac{\mathrm{Pyruvate}}{K_{b_{12}}} + \frac{\mathrm{NADH}}{K_{a_{12}}} + \frac{\mathrm{Pyruvate} \cdot \mathrm{NADH}}{K_{a_{12}} \cdot K_{b_{12}} \cdot \alpha_{12}}}$$

$$r13 = \frac{V_{m_{13}} \cdot pH_{inhibition} \cdot \mathrm{G6PD}}{K_{m_{13}} + \mathrm{G6P}} \cdot \mathrm{G6P}$$

$$r14 = k_{14} \cdot \mathrm{ATP}$$

$$r15 = k_{1_{15}} \cdot \mathrm{AMP} \cdot \mathrm{ATP} - k_{2_{15}} \cdot \mathrm{ADP}^2$$

$$r16 = pH_{inhibition} \cdot \left(V_{f_{16}} \cdot \mathrm{PFKFB2} \cdot \mathrm{F6P} - \frac{V_{r_{16}} \cdot \mathrm{PFKFB2} \cdot \mathrm{F26BP}}{K_{mp_{16}} \cdot K_{ms_{16}} + K_{mp_{16}} \cdot \mathrm{F6P} + K_{ms_{16}} \cdot \mathrm{F26BP}} \right)$$

$$r17 = \frac{V_{m_{17}} \cdot \mathrm{PHGDH} \cdot \mathrm{3PG}}{K_{m_{17}} + \mathrm{3PG}}$$

$$r18 = \frac{O_2 \cdot V_{m_{18}} \cdot \text{PDH} \cdot \text{Pyruvate}}{\left(K_{m_{18}} + \text{Pyruvate}\right) \cdot (O_2 + 0{,}011)}$$

$$r19 = \frac{V_{m_{19}} \cdot \text{ACC} \cdot \text{Citrate}}{K_{m_{19}} + \text{Citrate}}$$

$$r20 = \frac{V_{m_{20}} \cdot \text{SOD} \cdot \text{ROS}}{K_{m_{20}} + \text{ROS}}$$

$$r21 = V_{mf_{21}} \cdot \left(r21_{forward} - r21_{reverse}\right)$$

$$\text{r21}_{\text{forward}} = \frac{1}{e^{2\cdot(\text{Lactate}_{\text{out}} - Lactate)} + 1} \cdot \frac{Lactate}{Lactate + 0.01} \cdot \text{Regulation}_{\text{lac/pyr}}$$

$$\text{r21}_{\text{reverse}} = 0.2 \cdot \left(1 - \frac{1}{e^{2\cdot(\text{Lactate}_{\text{out}} - Lactate)} + 1}\right) \cdot \frac{\text{Lactate}_{\text{out}}}{\text{Lactate}_{\text{out}} + 0.1} \cdot \text{Regulation}_{\text{pH}_\text{e}/\text{pH}_\text{i}}$$

$$\text{Regulation}_{\text{lac/pyr}} = \frac{1}{1 + \frac{1}{\left(e^{Ratio_{lac/pyr} - 10}\right)^2}} \cdot \frac{1}{e^{150\cdot(0.1 - Pyruvate)} + 1}$$

$$\text{Ratio}_{\text{lac/pyr}} = \frac{Lactate + 0.001}{Pyruvate + 0.001}$$

$$\text{Regulation}_{\text{pH}_\text{e}/\text{pH}_\text{i}} = 1 - \frac{1}{e^{-2\cdot\left(pH_e - \frac{pH_i}{pH_{eq}}\right)} + 1}$$

$$r22 = k_{1_{22}} \cdot \text{R5P}^3 - k_{2_{22}} \cdot \text{F6P}^2 \cdot \text{G3P}$$

$$r23 = k_{23} \cdot pH_{inhibition} \cdot \text{R5P}$$

$$r24 = k_{24} \cdot \text{Serine}$$

$$r25 = \frac{O_2 \cdot k_{25} \cdot \text{NADH}}{O_2 + 0{,}011}$$

$$r26 = k_{26} \cdot \text{Citrate}$$

$$r27 = k_{27} \cdot \text{complex2}$$

$$r28 = O_2 \cdot k_{28} \cdot \text{complex2}$$

$$r29 = \frac{O_2 \cdot V_{m_{29}} \cdot \text{NOX}}{K_{m_{29}} + O_2}$$

$$r30 = k_{30} \cdot \text{Citrate}$$

$$pH_{inhibition} = 0.1 + \frac{0.9}{e^{\frac{6.7 - pH_e}{0.12}} + 1.0}$$
